# Supramolecular trap for catching polyamines in cells as an anti-tumor strategy

**DOI:** 10.1038/s41467-019-11553-7

**Published:** 2019-08-07

**Authors:** Junyi Chen, Hanzhi Ni, Zhao Meng, Jing Wang, Xiayang Huang, Yansheng Dong, Chao Sun, Yadan Zhang, Lei Cui, Jian Li, Xueshun Jia, Qingbin Meng, Chunju Li

**Affiliations:** 1 College of Environmental and Chemical Engineering, Shanghai University, Shanghai, 200444 P. R. China; 2 State Key Laboratory of Toxicology and Medical Countermeasures, Beijing Institute of Pharmacology and Toxicology, Beijing, 100850 P. R. China; 3 Department of Chemistry, Center for Supramolecular Chemistry and Catalysis, Shanghai University, Shanghai, 200444 P. R. China; 4 Key Laboratory of Inorganic-Organic Hybrid Functional Material Chemistry, Ministry of Education, Tianjin Key Laboratory of Structure and Performance for Functional Molecules, College of Chemistry, Tianjin Normal University, Tianjin, 300387 P. R. China

**Keywords:** Molecular capsules, Cancer therapy

## Abstract

Polyamines are essential for the growth of eukaryotic cells and can be dysregulated in tumors. Here we describe a strategy to deplete polyamines through host–guest encapsulation using a peptide-pillar[5]arene conjugate (P1P5A, P1 = RGDSK(N_3_)EEEE) as a supramolecular trap. The RGD in the peptide sequence allows the molecule to bind to integrin α_v_β_3_-overexpressing tumor cells. The negative charged glutamic acid residues enhance the inclusion affinities between the pillar[5]arene and cationic polyamines via electrostatic interactions and facilitate the solubility of the conjugate in aqueous media. The trap P1P5A efficiently encapsulates polyamines with association constants of 10^5^–10^6^ M^−1^. We show that P1P5A has a wide spectrum of antitumor activities, and induces apoptosis via affecting the polyamine biosynthetic pathway. Experiments in vivo show that P1P5A effectively inhibits the growth of breast adenocarcinoma xenografts in female nude mice. This work reveals an approach for suppressing tumor growth by using supramolecular macrocycles to trap polyamines in tumor cells.

## Abstract (web summary)

Polyamines are essential for cell growth and are frequently increased in concentration in cancer cells. Here, the authors use a macrocycle to generate a supramolecular trap, which depletes the polyamines in cells, induces apoptosis and reduces cancer cell growth in mice.

## Introduction

Polyamines, mainly including the aliphatic cations spermine (spm), spermidine (spd), and putrescine (put), exist in most living organisms and regulate a series of physiological processes including cell proliferation and differentiation^1–3^. Maintaining polyamine homeostasis by synergistic action between biosynthesis, catabolism and transport, is critical to cell survival^4,5^. Rapidly dividing cells like tumor cells require high amounts of polyamines to sustain tumor-related division^6,7^. Therefore, depleting polyamines to induce apoptosis of tumor cells has been identified as a feasible approach for the development of antitumor agents^8–10^. The construction of inhibitors targeting the polyamine biosynthetic pathway is one strategy that has been extensively studied^11,12^. The most representative inhibitor is 2-difluoromethylornithine (DMFO)^13,14^, which has been extended to Phase I–III clinical trials. Unfortunately, DMFO lacks clinical efficacy as a single agent in multiple clinical trials owing to its poor transport into cells and intracellular compensatory mechanisms^15–17^. Another methodology that has been reported is the use of polyamine analogs that are disparate enough from natural polyamines so that they can regulate physiological function, but not so different that they are not overly toxic to normal cells. However, it is very difficult to predict and control structural properties that favor both bioactivity and safety^18–20^.

**Fig. 1:**
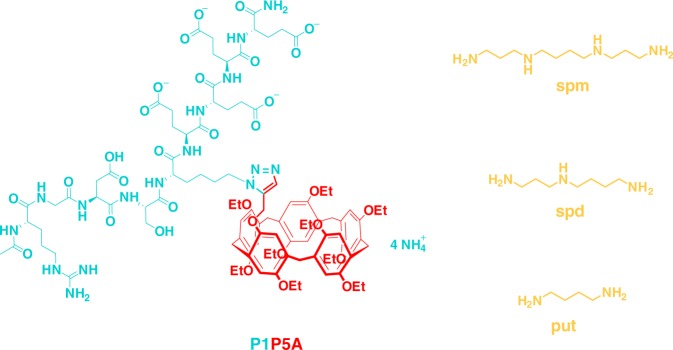
Chemical structures of P1P5A and polyamines

Herein, we propose a strategy for tumor treatment, in which polyamines are excessively depleted through host–guest encapsulation. Pillar[*n*]arenes^21^, an emerging type of macrocyclic hosts with a unique rigid and intrinsic symmetrical structures, has an outstanding ability to bind various kinds of guest molecules in a selective manner^22–29^. With a diameter of ~4.7 Å^30^, pillar[5]arene is an ideal host for linear spm, spd, and put due to their good matches in size and shape^31–33^. To deliver pillar[5]arene into tumor cells, the peptide arginine–glycine–aspartate (RGD) was added as a substituent onto the macrocycle. This modification was made because integrin, the most common receptor usually overexpressed on tumor cells^34–36^, specifically recognizes and binds to the RGD sequence, thus initiating integrin-mediated specific endocytosis^37^. As detailed below, our designed peptide-pillar[5]arene conjugate P1P5A (P1 = RGDSK(N_3_)EEEE) could catch polyamines, just like a supramolecular trap, to induce cell apoptosis (Fig. 1). The cytotoxicity against several types of classic tumor cells in vitro, the mechanism of cell apoptosis, and the tumor growth-inhibiting activity in vivo of P1P5A were comprehensively investigated. Although macrocycle-based vehicles, such as vesicles, nanoparticles, nanovalves, and direct host–guest complexation, for drug delivery have been widely documented^38–46^, the supramolecular trap P1P5A is unique because it can act by itself as a therapeutic agent by suppressing tumor growth.

## Results

### Design of supramolecular trap and host–guest complexation

The peptide-pillar[5]arene conjugates were designed, synthesized and characterized (Supplementary Figs. 1–17 and Supplementary Table 1), among which the peptide sequence of P1P5A was RGDSK(N_3_)EEEE (P1). The RGD sequence was chosen because it is a known target of integrin *α*_v_*β*_3_, which is widely expressed on tumor cells. Therefore cell-specific internalization of P1P5A should occur via ligand/receptor-mediated endocytosis. Azide-substituted lysine was conjugated with pillar[5]arene through copper(I)-catalyzed Huisgen alkyne–azide 1,3-dipolar cycloaddition (CuAAC “click” reaction). In addition, four anionic glutamic acid residues were added to enhance the molecule’s complexation ability to polyamines and to facilitate solubility. The pillar[5]arene segment as the effector molecule provided the ability to encapsulate polyamines via host–guest interactions.

**Fig. 2:**
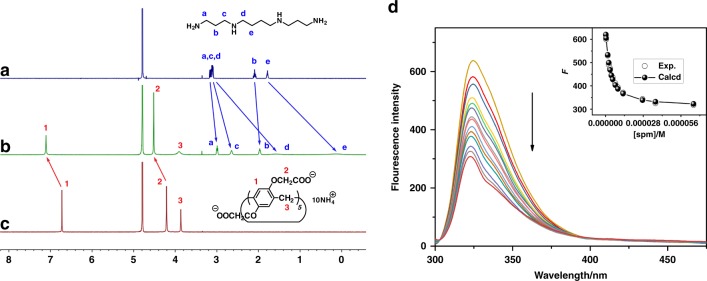
Host–guest complexation studies. ^1^H NMR spectra (500 MHz, 298 K, D_2_O) of **a** spm (3.0 mM), **b** spm (3.0 mM) + CP5A (3.0 mM) and **c** CP5A (3.0 mM); **d** fluorescence spectra of P1P5A (2.46 × 10^−6^ M) in aqueous PBS (pH 7.4) with different concentrations of spm at 298 K, inset: the nonlinear least-squares analysis to calculate the *K*_a_ value

First, the host–guest complexation behaviors between P1P5A and three polyamines (spm, spd, and put) were studied by ^1^H NMR spectroscopy. Although both P1P5A and the polyamines have good water solubility, the host–guest mixture was precipitated, indicating their strong interactions. No precipitate was found when mixing the three polyamines with carboxylatopillar[5]arene (CP5A), a widely used water-soluble pillar[5]arene. Figure 2a–c shows the spectra of spm in D_2_O recorded in the absence and presence of one equivalent of CP5A at 298 K. The proton signals of spm, especially for the middle methylenes H_d_ and H_e_, underwent substantial upfield shifts (Δ*δ* = −1.53 and −1.68 ppm for H_d_ and H_e_, respectively) and experienced considerable broadening upon the addition of CP5A. When the spectra were recorded above 313 K, a reduction in the spectral broadening of protons H_d_ and H_e_ was observed (Supplementary Fig. 18). Such spectral differences, which reflect complexation-induced shielding effects, are consistent with the formation of an inclusion complex and indicate that the middle methylene part (H_d_ and H_e_) is located in the cavity of pillar[5]arene. In addition, the peaks for the CP5A host shifted downfield due to complexation-induced deshielding effects. From 2D NOESY analysis, NOE correlations were observed between H_a_, H_b_, H_c_, and H_d_ of spm and the aromatic protons H_1_ of CP5A, as well as those between methylenes H_2_ located at the cavity portals of the host and the outer protons H_a_, H_b_, and H_c_ of spm (Supplementary Fig. 19). Both the ^1^H NMR and 2D NOESY results confirmed the formation of inclusion structures. Similar ^1^H NMR spectral changes were observed for spd and put mixed with CP5A (Supplementary Figs. 20 and 21), also demonstrating host–guest encapsulation. Controlled ^1^H NMR experiments of the mixtures of P1 moiety and the three polyamines were also performed. No precipitate was observed by mixing P1 and polyamines. In the presence of P1, the polyamine signals exhibit small shifts, less than 0.05 ppm, suggesting their weak interactions (Supplementary Figs. 22–24). These results further indicate the host–guest inclusion of P1P5A and polyamines.

To assess the host–guest encapsulation between P1P5A and the three polyamines quantitatively, fluorescence titration experiments were performed at 298 K in phosphate-buffered solution (PBS), pH 7.4. Upon the addition of spm, spd and put, respectively, the fluorescence intensity of P1P5A gradually decreased due to photo-induced electron transfer^47^. P1P5A exhibited strong binding affinities toward spm, spd, and put, with association constants (*K*_a_) of (3.98 ± 0.68) × 10^5^ M^−1^, (1.45 ± 0.14) × 10^5^ M^−1^, and (2.32 ± 0.68) × 10^6^ M^−1^, respectively (Fig. 2d, Supplementary Figs. 25 and 26). The above NMR and fluorescence titration experiments indicated the host–guest inclusion of polyamines, with a strong stability. Because the P1P5A supramolecular trap designed for catching polyamines in tumor cells was expected to be chemotherapy agents, we then conducted a series of related biological evaluations.

### Cytotoxicity of P1P5A

To examine the effect of P1P5A on cell viability, several tumor and normal cell lines were treated with various concentrations of P1P5A for 48 h, and then the cell viability was measured using a Cell Counting Kit-8 (CCK-8) assay. Concentration-dependent cell death was observed for all cell lines with the half-maximum inhibitory concentration (IC_50_) values ranging from 8 to 53 μM (Table 1). It was hypothesized that the capture of polyamines in an inert cavity of pillar[5]arene would disturb their normal physiological effects, and that excessive depletion of polyamines would cause cell death. No cytotoxic activity was observed for peptide P1, CP5A, or a 1:1 physical mixture of them even up to concentrations of 100 μM (see Supplementary Fig. 27), indicating that neither the peptide nor pillar[5]arene alone was responsible for the cytotoxicity and that the RGD sequence greatly promoted the trapping of P1P5A into the cells. To further confirm this finding, two control conjugates (P2P5A and P3P5A), in which the RGD sequence was replaced by GGD and RGE, respectively (see Supplementary Figs. 9, 12–15 and Supplementary Table 1), were synthesized. As expected, they did not effectively inhibit cell growth in any cell lines (Table 1, Supplementary Fig. 27). It was reasonable that GGD and RGE could not effectively increase the conjugate uptake. The RGD peptide sequence, as an essential and highly conserved component, has been demonstrated to bind with high affinity to integrin *α*_*v*_*β*_3_, thus facilitating the entry of the conjugate into cells better than the GGD or RGE sequence.

**Table 1:** IC_50_ values of P1P5A against selected cell lines (μM)

Tumor types	Cell lines	IC_50_ (μM)		
		P1P5A	P2P5A	P3P5A
Breast cancer	MCF-7	18.40	92.06	58.69
Glioma	C6	30.16	>100	>100
	U87	22.53	>100	73.43
Neuroma	Neuro-2a	8.52	79.75	45.07
Lung cancer	A549	34.81	>100	>100
Hepatocellular carcinoma	HepG-2	30.85	>100	>100
Normal	LO2	49.17	>100	>100
	HUVEC	52.15	>100	>100

### Induction of apoptosis

**Fig. 3:**
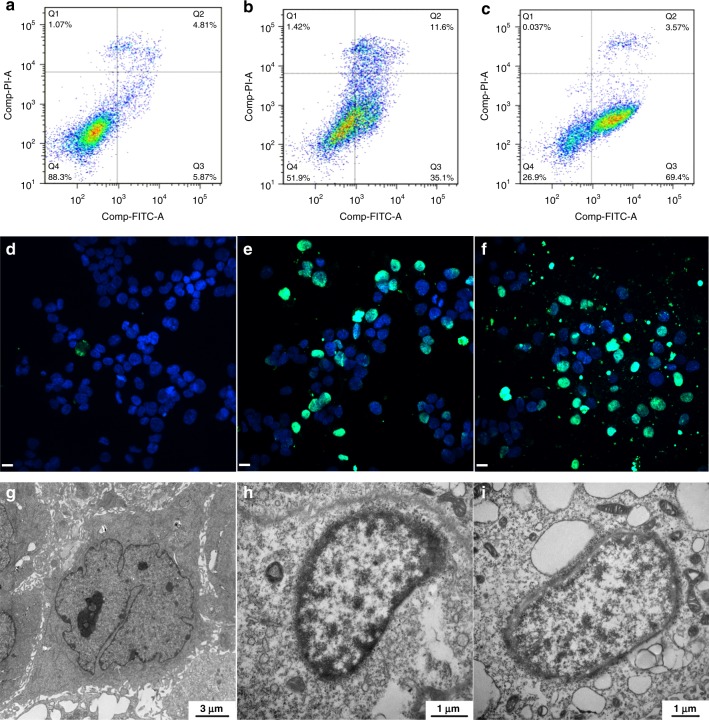
Confirmation of cell apoptosis. Contour diagram of Annexin V-FITC/PI flow cytometry results of MCF-7 cells incubated for 48 h. **a** Cells treated with PBS were used as a negative control; **b** cells treated with 25 μM P1P5A; **c** cells treated with 50 μM P1P5A. TUNEL analysis of MCF-7 cells incubated for 48 h. The apoptotic cells were labeled with FITC, while the nuclei were stained with DAPI; **d** cells treated with PBS were used as a negative control; **e** cells treated with 25 μM P1P5A; **f** cells treated with 50 μM P1P5A (scale bar: 24 μm). TEM images of ultrathin sections of MCF-7 cells incubated for 48 h; **g** cells treated with PBS were used as a negative control; **h** the cells treated with 25 μM P1P5A showed condensed chromatin and disappeared organelles; **i** the cells treated with 50 μM P1P5A showed the formation of many blebs and swollen or hollow mitochondria

Previous studies have shown that dysregulation of polyamine homeostasis can lead to apoptosis^7–10^. In order to determine whether the synthetic supramolecular trap can induce programmed cell death or necrosis, the FITC-Annexin V/propidium iodide (PI) method was employed in the human breast adenocarcinoma cell line MCF-7. As shown in Fig. 3a, after incubation for 48 h, negligible apoptosis happened in the PBS group. However, the percentage of apoptotic cells (including early and late apoptotic cells) in the group treated with 25 μM P1P5A was about 46.7%, while that in the presence of 50 μM P1P5A was significantly higher at 73.0% (Fig. 3b, c). These results clearly indicate that P1P5A could effectively induce the apoptosis of tumor cells and that the apoptosis rate depended on the dosage. Moreover, when seven other cell lines were treated with P1P5A, similar regulation of cell apoptosis was observed (Supplementary Fig. 28).

To further prove the occurrence of apoptosis, a commercially available terminal deoxynucleotidyl transferase dUTP nick end labeling (TUNEL) kit was used to observe cells treated with different doses of P1P5A. The TUNEL staining assay displayed that P1P5A could induce severe apoptosis of MCF-7 cells (green dots) in both dosage groups (Fig. 3e, f) compared with almost no apoptosis in the control group (Fig. 3d). These results were in accordance with the flow cytometry results, revealing that P1P5A effectively inhibited tumor cell growth by inducing apoptosis.

Furthermore, transmission electron microscopy (TEM) was used to directly view the ultramorphological changes of MCF-7 cells treated with P1P5A. As shown in Fig. 3h, i, the cells treated with P1P5A were apoptotic with a changed morphology, condensed chromatin, and damaged or disappeared organelles; while the control cells presented a normal structure (Fig. 3g). The apoptotic process started with the condensation of chromatin at the nuclear periphery, then many blebs were generated, and finally swollen or hollow mitochondria emerged (Fig. 3i).

### Molecular mechanism

**Fig. 4:**
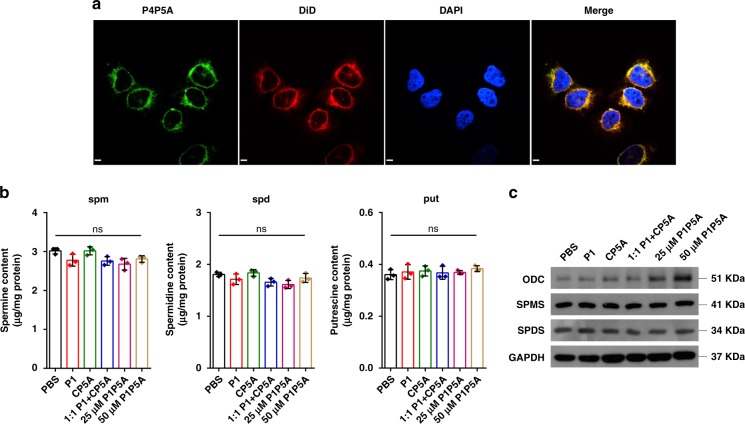
Investigation of the molecular mechanism. **a** CLSM images of MCF-7 cells after incubation with 50 μM P4P5A for 48 h. For each panel, the images from left to right show NBD fluorescence in cells (green), cell membranes stained by DiD (red), cell nuclei stained by DAPI (blue), and overlays of the three images (scale bar: 20 μm); **b** HPLC analysis was used to detect the intracellular concentration in MCF-7 (*n* = 3, mean ± SD); **c** Western blot analysis of enzymes associated with polyamine metabolism in MCF-7 cells treated with various compounds for 6 h; GAPDH was used as an internal control. Significant differences were assessed in (**b**) using the *t* test. ns not significant

Confocal laser scanning microscopy (CLSM) was conducted to verify the distribution of the supramolecular traps in cells. 7-Fluoro-4-nitro-2,1,3-benzoxadiazole (NBD-F), a commercially available fluorescent labeling reagent (green fluorescence), was used to label P1P5A via the amino group of the additional lysine residue at the C-terminus to obtain the conjugate P4P5A (Supplementary Figs. 9, 16, 17, and Supplementary Table 1). As shown in Fig. 4a, 4’,6-diamidino-2-phenylindole (DAPI) was used to stain the cell nucleus blue; and 1,1’-dioctadecyl-3,3,3’,3’-tetramethylindodicarbocyanine (DiD), a lipophilic fluorescent dye, was used to stain cell membranes and other fat-soluble biological structures red. MCF-7 cells exhibited strong intracellular green fluorescence after incubation with 50 μM P4P5A for 48 h, and green dots corresponding to P4P5A colocalized with DiD, revealing an obvious internalizing behavior and concentrated distribution in the cytoplasm. The above results showed that the conjugate was mainly enriched in the cytoplasm and did not enter the nuclei of MCF-7 cells.

Having confirmed the distribution of the conjugates in the cytoplasm, we evaluated the effect of P1P5A on intracellular polyamine depletion. As shown in Fig. 4b, P1P5A treatment for 6 h did not change the intracellular concentration of spm, spd, or put in MCF-7 cells. This result was quite reasonable because the host–guest inclusion did not change the polyamine structures. Meanwhile, by western blot analysis, we measured the contents of ornithine decarboxylase (ODC), spermidine synthase (SPDS), and spermine synthase (SPMS) which generate the corresponding polyamines. The first rate-limiting step of polyamine biosynthesis is that ornithine generates put catalyzed by ODC. SPDS and SPMS, specific aminopropyl transferases, sequentially produce spd from put and spm from spd, respectively (see Supplementary Fig. 29). When the P1P5A traps catch these polyamines to form the host–guest complexes, more enzymes would be expressed to assist the production of more spm, spd, and put as feedbacks. As shown in Fig. 4c, the expression levels of ODC in cells treated with P1P5A were much higher than those treated with PBS, P1, CP5A, and 1:1 P1 + CP5A. The gray-scale results display a dose-dependent effect between the low- and high-dose groups. The contents of ODC reflected the consumption of put due to compensatory mechanism. In contrast, treatment with P1P5A did not induce increase in expression of SPDS and SPMS, which is reasonable since SPDS and SPMS are constitutively expressed and are not easily induced by external factors^10^. The effect of host–guest interaction, although did not change the chemical structure of these polyamine molecules, merely affected the normal physiological activities of spm, spd, and put through inclusion in the host cavity.

### In vivo tumor inhibition studies

**Fig. 5:**
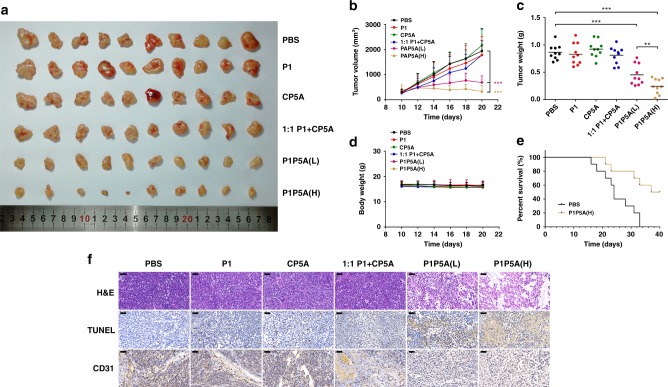
In vivo antitumor experiments. **a** Images of tumors excised from MCF-7 xenograft nude mice treated with PBS (control), free P1, free CP5A, a 1:1 mixture of P1 and CP5A, low-dose P1P5A (L), and high-dose P1P5A (H); **b** Tumor growth curves of the MCF-7 tumor model in the groups treated with PBS, P1, CP5A, 1:1 P1 + CP5A, P1P5A (L), and P1P5A (H), mean ± SD (*n* = 10 mice per groups); **c** normalized tumor weights at 10 days after treatment with PBS, P1, CP5A, 1:1 P1 + CP5A, P1P5A (L), and P1P5A (H), mean ± SD (*n* = 10 mice per group); **d** changes in body weight observed for MCF-7 xenograft nude mice after treatment with PBS, P1, CP5A, 1:1 P1 + CP5A, P1P5A (L), and P1P5A (H), mean ± SD (*n* = 10 mice per group); **e** survival rates of nude mice bearing MCF-7 tumors treated with PBS and P1P5A (H) (*n* = 10 mice per group); **f** H&E, TUNEL, and CD31 analyses of tumor tissue at 10 days after treatment with PBS, P1, CP5A, 1:1 P1 + CP5A, P1P5A (L), and P1P5A (H) (scale bar: 50 μm). Significant differences were assessed in (**b**) and (**c**) using the *t* test. **p < 0.01, ***p < 0.001

To assess the efficacy of P1P5A in vivo, subcutaneous tumor xenograft mouse models were used. The results (Fig. 4a, b) revealed that the tumor volumes in the PBS, P1, CP5A, and 1:1 mixture of P1 and CP5A groups increased about sixfold over the original state after 10 days, while both P1P5A groups, 100 mg kg^−1^ (L) and 200 mg kg^−1^ (H) per time, exhibited a remarkable inhibitory effect on tumor growth. The tumor volume was much less in the high-dose P1P5A group than in the low-dose group. The final tumor weight was measured to further validate the efficacy of P1P5A (Fig. 5c). The normalized tumor weight of the PBS group (0.87 ± 0.14 g) was 93% higher than that of the low-dose P1P5A group (0.45 ± 0.19 g) and 260% higher than that of the high-dose group (0.24 ± 0.14 g). The 1:1 mixture of P1 and CP5A had no antitumor activity. In addition, we also tried three lower doses of P1P5A, 25, 50, and 75 mg kg^−1^. Although the P1P5A groups of 25 and 50 mg kg^−1^ had no obvious inhibitory effect on tumor growth, the 75 mg kg^−1^ group embodied certain antitumor activity (Supplementary Fig. 30). Taken together, these results indicated that P1P5A could efficiently suppress tumor growth and that the covalent linkage of the peptide and pillar[5]arene macrocycle played a decisive role in the antitumor activity.

In addition to evaluating the therapeutic effect with different treatments, hematoxylin and eosin (H&E), TUNEL, and CD31 staining assays were performed to detect apoptotic cells and inhibition of angiogenesis in tumor tissue (Fig. 5f). The images of H&E-stained tumor tissues showed the lack of discernible boundary regions and the absence of many nuclei, indicating that the two doses of P1P5A could induce necrosis. Compared to the other groups, the P1P5A groups showed the highest number of TUNEL-positive tumor cells, indicating massive apoptosis. In addition, the CD31-stained immunohistochemical sections of the tumors showed that the P1P5A groups had the least angiogenesis, confirming that the treatment efficiency inhibited cell proliferation in the tumor tissues.

For antitumor therapeutic agents, safety in vivo is very important. Therefore, changes in body weight and the survival rate of the tumor-bearing mice after treatment were determined to reflect the systematic toxicity. As shown in Fig. 5d, the body weights of the mice in all groups remained almost the same as that of the control group, demonstrating that P1P5A showed a negligible effect on body weight. In addition, Kaplan–Meier analyses showed that the median survival of the mice treated with PBS was 24 days; for P1P5A, it was significantly prolonged to over 36 days (Fig. 5e). To further prove the low systemic toxicity of P1P5A for tumor therapy, the main organs including the heart, liver, spleen, lung, and kidney of the mice with different treatments were harvested and analyzed by H&E staining at 10 days post operation. The results of the histological analysis showed no significant inflammatory or toxicological responses in the control groups (P1, CP5A, 1:1 P1 + CP5A) or the P1P5A administration groups, compared to the PBS group (Supplementary Fig. 31). These results indicate that P1P5A was well-tolerated in the major organs.

## Discussion

In summary, we have described a supramolecular therapeutic agent P1P5A as an inhibitor of tumor growth. The pillar[5]arene molecular container could trap polyamines, restrict their normal physiological effects, and therefore induces cell apoptosis. Particularly, the peptide segment containing the RGD sequence played a crucial role in the activity of the compound, due to its efficient binding to integrin *α*_*v*_*β*_3_ and mediation of specific endocytosis. P1P5A could inhibit several kinds of tumor cells via a polyamine-dependent pathway in in vitro experiments. In addition, in vivo experiments also confirmed the antitumor efficiency of P1P5A that it effectively inhibited the growth of MCF-7 xenografts in female nude mice, together with a high median survival and a low systemic toxicity. Overall, this work proposed a strategy that macrocyclic derivatives can perform as antitumor agents instead of only as supramolecular vehicles for drug delivery. This research might pave a way to win the battle against cancer.

## Methods

### General

All reagents were purchased commercially and used without further purification unless otherwise noted. CP5A was synthesized and purified according to previous reports. *N*-Fluorenyl-9-methoxycarbonyl (Fmoc)-protected l-amino acids were purchased from GL Biochem Ltd. (Shanghai, China). Rink amide resin was purchased from Nankai Hecheng (Tianjin, China). Roswell Park Memorial Institute (RPMI) 1640 medium, minimum Eagle’s medium (MEM), and Dulbecco’s modified Eagle medium (DMEM) were purchased from Gibco (Thermo Fisher Scientific). Fetal bovine serum (FBS), penicillin–streptomycin and PBS were purchased from Invitrogen (Carlsbad, CA, USA). The Cell Counting Kit-8 (CCK-8) was purchased from Dojindo China Co. Ltd. (Shanghai, China). The Annexin V-FITC/PI apoptosis detection kit was purchased from Zoman Biotechnology Co. Ltd. (Beijing, China). MCF-7, C6, U87, Neuro-2a, A549, HepG-2, LO2, and HUVEC were purchased from Cell Resource Center (Beijing, China). Anti-ODC antibody (ab66067), anti-spermidine synthase antibody (ab111884), and anti-spermine synthase antibody (ab156879) were all purchased from Abcam Co. Ltd. (Shanghai, China). ^1^H NMR, ^13^C NMR, and 2D NOESY spectra were recorded using a JNM-ECA-400 or a Bruker Advance 500 MHz spectrometer. High-resolution mass spectra were recorded on a Bruker Daltonics, Inc. APEXIII 7.0 TESLA FTMS instrument and a Bruker Daltonics, Inc. APEX Qe FT-ICRMS instrument.

### Synthesis and characterization

Synthesis and relevant characterization details are provided in the Supplementary Information.

### Determination of the association constants

To quantitatively assess the inclusion complexation behavior of these compounds, fluorescence titrations of P1P5A with spm, spd, and put were performed at 298 K in a phosphate buffer solution of pH 7.4. Using a nonlinear least-squares curve-fitting method, the association constant was obtained for each host–guest combination from the following equation

$$F = \, I_{0} - ( 0.5 \ast ( {\mathrm{alpha}} ) \ast (([H]_{0}/2 + [G] + (1/K_{a})) - ({\mathrm{sqrt}} ( ( [H]_0/2 + [G] \\ + \, ( {1/K_a} ) ) \ast ([H]_{0}/2 + [G] + (1/K_{a})) - 4 \ast [ H ]_{0}/2^\ast [G])))),$$

where *F* is the fluorescence intensity at [*G*], *I*_0_ is the initial fluorescence intensity of the host alone, [*H*]_0_ is the fixed initial concentration of the host, and [*G*] is the concentration of guest. Typical fluorescence spectrum changes and curve-fitting plots are shown in Fig. 2d and Supplementary Figs. 25 and 26.

### In vitro cell assay

The relative in vitro cytotoxicity of P1P5A against all cell lines was assessed using the CCK-8, according to the manufacturer’s instructions. To test P1P5A, MCF-7, HepG-2, and A549 cells were seeded into 96-well plates at a density of 5000 cells/well in 100 µL of RPMI 1640 medium containing 10% FBS, 1% penicillin, and 1% streptomycin. C6, U87, and Neuro-2a cells were seeded into 96-well plates at a density of 5000 cells/well in 100 µL of MEM supplemented with 10% FBS, 1% penicillin, and 1% streptomycin. Likewise, LO2 and HUVEC cells were seeded into 96-well plates at a density of 5000 cells/well in 100 µL of complete DMEM supplemented with 10% FBS, 1% penicillin, and 1% streptomycin and cultured for 24 h in 5% CO_2_ at 37 °C. P1P5A was dissolved in PBS and then diluted to the required concentration. It was then added to the cell-containing wells and further incubated at 37 °C under 5% CO_2_ for 48 h. Subsequently, 10 µL of CCK-8 was added into each well and incubated for another 1 h. The plates were then measured at 450 nm using a SpectraMax® M5 plate reader (Molecular Devices, San Jose, CA, USA). All experiments were carried out five times. For P1, CP5A, and the 1:1 mixture of P1 and CP5A, the same procedures were performed by varying the concentration of the species in question to determine the cytotoxicity.

### Cell apoptosis

All cell lines were seeded into 6-well plates at a density of 150,000 cells/well in 1.5 mL of medium supplemented with 10% FBS, 1% penicillin, and 1% streptomycin and cultured for 24 h in 5% CO_2_ at 37 °C. P1P5A was dissolved in PBS and further incubated at 37 °C under 5% CO_2_ for 48 h. After 48 h, the cells were harvested and washed three times with cold PBS and resuspended in 500 μL of binding buffer. Then 5 μL of Annexin V-FITC and 5 μL of PI were added and incubated with the cells for 15 min in the dark. Finally, the stained cells were analyzed by a flow cytometer.

### TUNEL staining assay

For TUNEL staining, MCF-7 cells were seeded into 6-well plates at a density of 1 × 10^5^ cells/well in 1.5 mL of RPMI 1640 medium supplemented with 10% FBS, 1% penicillin, and 1% streptomycin and cultured for 24 h in 5% CO_2_ at 37 °C. P1P5A was dissolved in PBS and further incubated at 37 °C under 5% CO_2_ for 48 h. After treatment, the cells were collected, fixed, permeabilized, and then subjected to the TUNEL assay, according to the instructions provided by the Apoptosis Assay Kit (Roche, China). Nuclei were stained by DAPI dye after TUNEL staining. Afterwards, the samples were observed with a laser-scanning confocal microscope (LSM 510 META, Carl Zeiss, Germany).

### Cellular ultrastructure observation by TEM

The ultrastructural alterations of MCF-7 cells induced by pristine grapheme were observed with a Hitachi TEM (Hitachi H-7650, Japan) at an accelerating voltage of 80 kV. MCF-7 cells were seeded into 6-well plates at a density of 1 × 10^5^ cells/well in 1.5 mL of RPMI 1640 medium supplemented with 10% FBS, 1% penicillin, and 1% streptomycin, and cultured for 24 h under 5% CO_2_ at 37 °C. P1P5A was dissolved in PBS and further incubated at 37 °C under 5% CO_2_ for 48 h. After treatment, the cells were collected and fixed overnight with 2.5% glutaraldehyde solution in PBS at 4 °C for 2 h. The fixed cells were washed several times with PBS and post-fixed with 1% osmic acid in PBS for 1 h. After washing three times with PBS at 4 °C, the fixed samples were dehydrated with a series of graded ethanol and acetone solutions, incubated with a mixture of acetone and Epon812 resin (1:1, v/v) for 30 min at 4 °C, and then incubated with Epon812 resin overnight. Next, the samples were embedded in Epon812 resin, and ultrathin sections were obtained (60–70 nm) with an ULTRACUTS Ultramicrotome (RMC, USA). Lastly, the sections were stained with uranyl acetate and lead citrate for 10 min, respectively, before observation by TEM.

### Confocal laser scanning microscopy

MCF-7 cells were seeded into 6-well plates at a density of 1 × 10^5^ cells/well in 1.5 mL of RPMI 1640 medium supplemented with 10% FBS, 1% penicillin, and 1% streptomycin and cultured for 24 h under 5% CO_2_ at 37 °C. A solution of 50 μM P4P5A was added to each well, and the plate was further incubated at 37 °C under 5% CO_2_ for 48 h. After treatment, the cells were washed three times with PBS. The cell membranes were stained with DiD solution (10 μM) for 20 min and washed three times with PBS. Then, the cells were fixed with 4% paraformaldehyde for 10 min at room temperature. The cells were carefully washed three times with PBS after immobilization. The cell nuclei were stained with DAPI for 20 min and then washed three times with PBS. The cellular uptake was observed by CLSM (LSM 510 META, Carl Zeiss, Germany).

### Analysis of intracellular polyamines

Cells were seeded into 6-well plates at 1 × 10^5^ cells/well and cultured for 24 h under 5% CO_2_ at 37 °C. Then the serum-containing medium was removed, and cells were rinsed twice with PBS. Further cells were cultured with a serum-free medium containing various compounds for 6 h. After treatment, the cells were harvested by trypsinization, and then were resuspended in PBS. A part of cell suspension was used to determine the protein content according to the BCA method, and another part of the suspension was add with perchlorate solution to obtain cell extractive. The cell extractive was mixed with hexanediamine and dansyl chloride, and pH was adjusted to 9.5 with saturated sodium carbonate solution, reacting at 50 °C for 30 min. After cooling, ethyl acetate was added to extract the sample solution, and the concentrations of the natural polyamines (spm, spd, and put) were determined by reversed-phase high-performance liquid chromatography.

### Western blotting analysis

MCF-7 cells were seeded into 6-well plates at 1 × 10^5^ cells/well and cultured for 24 h under 5% CO_2_ at 37 °C. Then the serum-containing medium was removed, and cells were rinsed twice with PBS. Further cells were cultured with a serum-free medium containing various compounds for 6 h at 37 °C under 5% CO_2_. Following incubation, cells were washed with PBS, harvested with a scraper and centrifuged at 12 K rpm for 15 min at 4 °C. Discarding the supernatant, cells were lysed to collect the cellular protein. Equal amounts of proteins were separated by 10% sodium dodecyl sulfate–polyacrylamide gel electrophoresis and transferred to polyvinylidene difluoride (PVDF) membranes. The membranes were blocked with 5% nonfat milk in Tris-buffered saline containing Tween 20 at room temperature and then incubated with rabbit anti-spermine synthase antibody (1:1000), rabbit anti-spermidine synthase antibody (1:1000), mouse anti-ODC antibody (1:1000), and mouse anti-GAPDH antibody (1:2000), respectively, overnight at 4 °C. The PVDF membranes were then incubated with horseradish peroxidase-conjugated goat anti-rabbit and goat anti-mouse immunoglobulin G antibody (1:5000), respectively, for 1 h at room temperature. Immunoreactive bands were visualized with a chemiluminescene substrate kit.

### In vivo antitumor efficacy

Six-week-old female Balb/C nude mice, weighing 15 g and purchased from the Beijing Experimental Animal Center (Beijing, China), were used. A total of 2 × 10^6^ MCF-7 cells in 200 μL of saline were inoculated subcutaneously into the right dorsal flanks of the mice. The mice were normally fed after inoculation for 10 days. In total, 60 mice were randomly divided into 6 groups: the PBS group, free P1 group, free CP5A group, 1:1 P1 + CP5A group (all equivalent to 200 mg kg^−1^ P1P5A), and two P1P5A groups (low-dose, L: 100 mg kg^−1^ and high-dose, H: 200 mg kg^−1^ per time). The mice were then treated through subcutaneous injection at different time points (on days 1, 3, 5, 7, and 9). The tumor volumes and the weights of the mice were recorded at different time points after treatment. The mice were sacrificed on day 11, and the tumors were separated from the animals and weighed. All experimental procedures were conducted in accordance with the Guide for the Care and Use of Laboratory Animals of the AAALAC, and were approved by the Animal Care and Use Committee of National Beijing Center for Drug Safety Evaluation and Research. Best efforts were made to minimize the number of animals used and their suffering.

### Reporting summary

Further information on research design is available in the Nature Research Reporting Summary linked to this article.

## Supplementary information

Supplementary Information

Reporting Summary

Source Data

## Data Availability

The authors declare that the data supporting the findings of this study are available within the paper and its Supplementary Information. All data are available from the authors on reasonable request.

## References

[1] Tabor CW, Tabor H (1984). Polyamines. Ann. Rev. Biochem..

[2] Pegg AE (1986). Recent advances in the biochemistry of polyamines in eukaryotes. Biochem. J..

[3] Pegg AE, Casero RA (2011). Current status of the polyamine research field. Methods Mol. Biol..

[4] Marton LJ, Pegg AE (1995). Polyamines as targets for therapeutic intervention. Annu. Rev. Pharmacol. Toxicl..

[5] Wallace HM, Fraser AV, Hughes A (2003). A perspective of polyamine metabolism. Biochem. J..

[6] Jänne J, Pösä H, Raina A (1978). Polyamines in rapid growth and cancer. Biochim. et. Biophys. Acta.

[7] Thomas T, Thomas TJ (2003). Polyamine metabolism and cancer. J. Cell. Mol. Med..

[8] Gerner EW, Meyskens FL (2004). Polyamines and cancer: old molecules, new understanding. Nat. Rev. Cancer.

[9] Seiler N, Raul F (2005). Polyamines and apoptosis. J. Cell. Mol. Med..

[10] Casero RA, Marton LJ (2007). Targeting polyamine metabolism and function in cancer and other hyperproliferative diseases. Nat. Rev. Drug Discov..

[11] Pegg AE (1988). Polyamine metabolism and its importance in neoplastic growth and as a target for chemotherapy. Cancer Res..

[12] Thomas T, Thomas TJ (2001). Polyamines in cell growth and cell death: molecular mechanisms and therapeutic applications. Cell. Mol. Life Sci..

[13] McCann PP, Pegg AE (1992). Ornithine decarboxylase as an enzyme target for therapy. Pharmac. Ther..

[14] Meyskens FL, Gerner EW (1999). Development of difluoromethylornithine (DFMO) as a chemoprevention agent. Clin. Cancer Res..

[15] Ganju V, Edmonson JH, Buckner JC (1994). Phase I study of combined alpha interferon, alpha difluoromethylornithine (DFMO), and doxorubicin in advanced malignancy. Invest. New Drug..

[16] Meyskens FL, Kingsley EM, Glattke T, Loescher L, Booth A (1986). A phase II study of α-difluoromethylornithine (DFMO) for the treatment of metastatic melanoma. Invest. New Drug..

[17] Levin VA (2003). Phase III randomized study of postradiotherapy chemotherapy with combination α-difluoromethylornithine-PCV *versus* PCV for anaplastic gliomas. Clin. Cancer Res..

[18] Bergeron RJ, Hawthorne TR, Timothy Vinson JR, Beck Jr. DE, Ingeno MJ (1989). Role of the methylene backbone in the antiproliferative activity of polyamine analogues on L1210 cells. Cancer Res..

[19] Ha HC, Woster PM, Casero RA (1998). Unsymmetrically substituted polyamine analogue induces caspase-independent programmed cell death in bcl-2-overexpressing cells. Cancer Res..

[20] Casero RA, Woster PM (2001). Terminally alkylated polyamine analogues as chemotherapeutic agents. J. Med. Chem..

[21] Ogoshi T, Kanai S, Fujinami S, Yamagishi T, Nakamoto Y (2008). *para*-Bridged symmetrical pillar[5]arene: their lewis acid catalyzed synthesis and host-guest property. J. Am. Chem. Soc..

[22] Ogoshi T, Yamagishi T, Nakamoto Y (2016). Pillar-shaped macrocyclic hosts pillar[*n*]arene: new key players for supramolecular chemistry. Chem. Rev..

[23] Yu G, Jie K, Huang F (2015). Supramolecular amphiphiles based on host-guest molecular recognition motifs. Chem. Rev..

[24] Li B (2017). A pH responsive complexation-based drug delivery system for oxaliplatin. Chem. Sci..

[25] Hao Q (2018). Supramolecular chemotherapy: carboxylated pillar[6]arene for decreasing cytotoxicity of oxaliplation to normal cells and improving its anticancer bioactivity against colorectal cancer. Acs Appl. Mater. Interfaces.

[26] Ni M (2016). Dramatically promoted swelling of a hydrogel by pillar[6]arene–ferrocene complexation with multistimuli responsiveness. J. Am. Chem. Soc..

[27] Song N, Kakuta T, Yamagishi T, Yang Y, Ogoshi T (2018). Molecular-scale porous materials based on pillar[n]arenes. Chem.

[28] Yang K, Pei Y, Wen J, Pei Z (2016). Recent advances in pillar[n]arenes: synthesis and applications based on host–guest interactions. Chem. Commun..

[29] Wu M (2018). Multistimuli responsive core–shell nanoplatform constructed from Fe_3_O_4_@MOF equipped with pillar[6]arene nanovalves. Small.

[30] Xue M, Yang Y, Chi X, Zhang Z, Huang F (2012). Pillararenes, a new class of macrocycles for supramolecular chemistry. Acc. Chem. Res..

[31] Zhang H, Liu Z, Zhao Y (2018). Pillararene-based self-assembled amphiphiles. Chem. Soc. Rev..

[32] Wang Y, Ping G, Li C (2016). Efficient complexation between pillar[5]arenes and neutral guests: from host-guest chemistry to functional materials. Chem. Commun..

[33] Li C (2013). Molecular selective binding of basic amino acids by a water-soluble pillar[5]arene. Chem. Commun..

[34] Pierschbacher MD, Ruoslahti E (1984). Cell attachment activity of fibronectin can be duplicated by small synthetic fragments of the molecule. Nature.

[35] Ruoslahti E, Pierschbacher MD (1987). New perspectives in cell adhesion: RGD and integrins. Science.

[36] Wang F (2013). The functions and applications of RGD in tumor therapy and tissue engineering. Int. J. Mol. Sci..

[37] Ruoslahti E (1996). RGD and other recognition sequences for integrins. Annu. Rev. Cell Dev. Biol..

[38] Ma D (2012). Acyclic cucurbit[n]uril molecular containers enhance the solubility and bioactivity of poorly soluble pharmaceuticals. Nat. Chem..

[39] Chen Y, Huang Z, Xu J, Sun Z, Zhang X (2016). Cytotoxicity regulated by host-guest interactions: a supramolecular strategy to realize controlled disguise and exposure. Acs Appl. Mater. Interfaces.

[40] Chen H (2018). Precise nanomedicine for intelligent therapy of cancer. Sci. China Chem..

[41] Ma X, Zhao Y (2015). Biomedical applications of supramolecular systems based on host−guest interactions. Chem. Rev..

[42] Guo D, Wang K, Wang Y, Liu Y (2012). Cholinesterase-responsive supramolecular vesicle. J. Am. Chem. Soc..

[43] Ko SK (2014). Synthetic ion transporters can induce apoptosis by facilitating chloride anion transport into cells. Nat. Chem..

[44] Chang Y (2014). Cationic vesicles based on amphiphilic pillar[5]arene capped with ferrocenium: a redox-responsive system for drug/siRNA co-delivery. Angew. Chem. Int. Ed..

[45] Zhou J, Yu G, Huang F (2017). Supramolecular chemotherapy based on host-guest molecular recognition: a novel strategy in the battle against cancer with a bright future. Chem. Soc. Rev..

[46] Angelos S, Yang Y, Patel K, Stoddart JF, Zink JI (2008). pH-Responsive supramolecular nanovalves based on cucurbit[6]uril pseudorotaxanes. Angew. Chem. Int. Ed..

[47] Silva, A. P. & Rupasinghe, R. A. D. D. A new class of fluorescent pH indicators based on photo-induced electron transfer. *J. Chem. Soc. Chem. Commun*. 1669–1670 (1985). 10.1039/C39850001669.

