# Supplementary material for: Supramolecular trap for catching polyamines in cells as an anti-tumor strategy
Source: Nat Commun. 2019 Aug 7;10:3546. doi: 10.1038/s41467-019-11553-7 (PMC6685945; doi:10.1038/s41467-019-11553-7)
Supplement: Supplementary file 1 — Supplementary Information [file 41467_2019_11553_MOESM1_ESM.pdf]

Supplementary Information

Supramolecular trap for catching polyamines in cells as an antitumor strategy

Junyi Chen, Hanzhi Ni, Zhao Meng, Jing Wang, Xiayang Huang, Yansheng Dong, Chao Sun, Yadan Zhang, Lei Cui, Jian Li, Xueshun Jia, Qingbin Meng,* and Chunju Li*

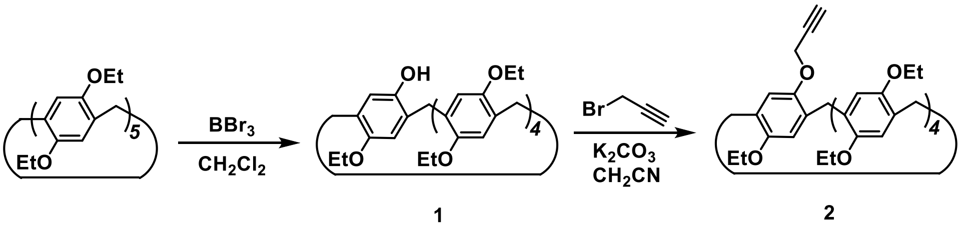

Supplementary Figure 1. The synthetic route to **2**.

Carboxylatopillar[5]arene (CP5A) was synthesized according to the literature methods[1].

Synthesis of 1. To a solution of per-ethylated pillar[5]arene (0.89 g, 1.0 mmol) in dichloromethane (50 mL), boron tribromide (0.084 mL, 0.90 mmol) was added under nitrogen atmosphere. The mixture was stirred at 25 °C for 1 h. Then, water was added into the mixture and the organic layer was dried with Na_2SO_4. After desiccation, the solution was poured into methanol and the resulting precipitate was collected by filtration. The residue was purified by column chromatography on silica gel (petroleum ether : ethyl acetate = 10 : 1) to afford a white solid (**1**, 0.21 g, 0.24 mmol, Yield: 24%). ^{1}H NMR (400 MHz, chloroform-*d*) δ (ppm): 6.90, 6.70, 6.68, 6.65, 6.63, 6.55, 6.54 (m, 10H), 4.06, 4.04, 4.02, 4.01, 3.93, 3.91, 3.90, 3.88, 3.86, 3.84, 3.82, 3.80, 3.78, 3.77, 3.76, 3.74, 3.73, 3.71, 3.65, 3.64, 3.62, 3.60 (m, 28H), 1.43, 1.41, 1.39, 1.37, 1.35, 1.24, 1.23, 1.21, 1.20, 1.18, 1.16, 1.15, 1.13, 1.11, 1.10, 1.08, 1.04, 1.03, 1.01 (m, 27H). ^{13}C NMR (100 MHz, chloroform-*d*) δ (ppm): 151.17, 150.30, 150.20, 150.11, 150.00, 149.97, 149.79, 147.55, 147.52, 130.43, 129.61, 128.93, 128.85, 128.82, 128.63, 128.46, 127.93, 127.11, 125.16, 119.20, 116.06, 115.81, 115.50, 115.39, 115.36, 115.20, 114.96, 113.72, 113.51, 65.13, 64.56, 64.32, 64.13, 63.99, 63.97, 63.88, 63.80, 31.17, 30.56, 30.21, 29.58, 29.32, 15.33, 15.19, 15.12, 15.05, 15.00, 14.80, 14.75, 14.73. HRMS (ESI): $C_{53}H_{67}O_{10}$ $[M]^+$. calcd m/z 863.4729; found m/z 863.4748.

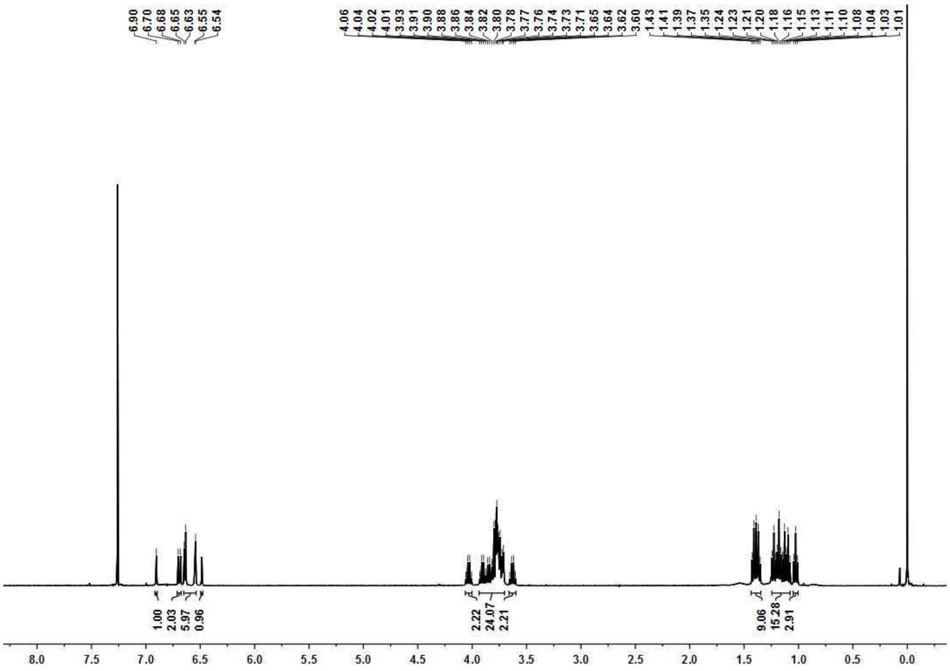

Supplementary Figure 2. ^{1}H NMR spectra (400 MHz, chloroform-*d*, 298 K) of **1**.

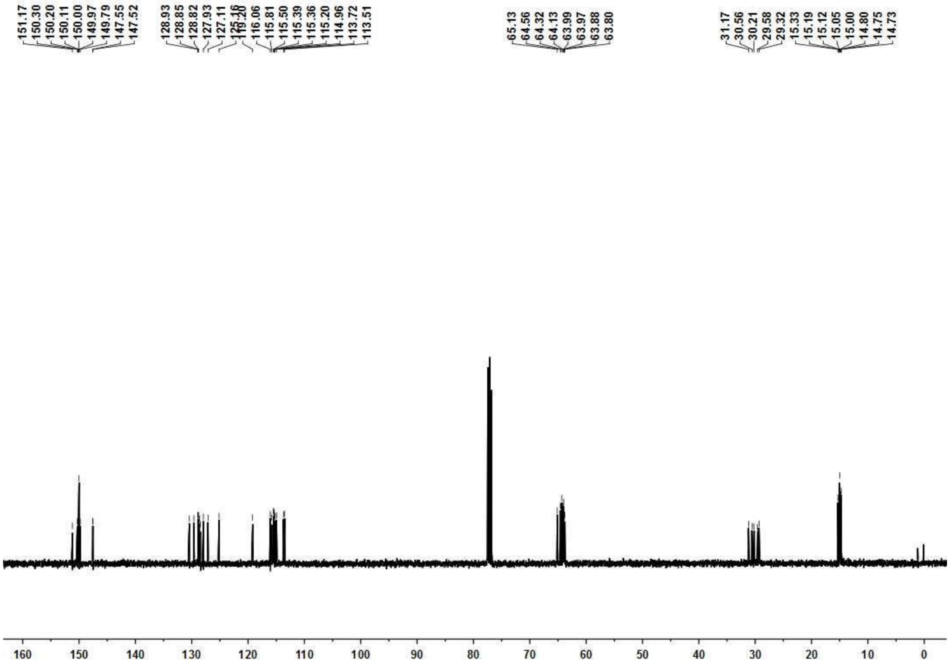

Supplementary Figure 3. ^{13}C NMR spectra (100 MHz, chloroform-*d*, 298 K) of **1**.

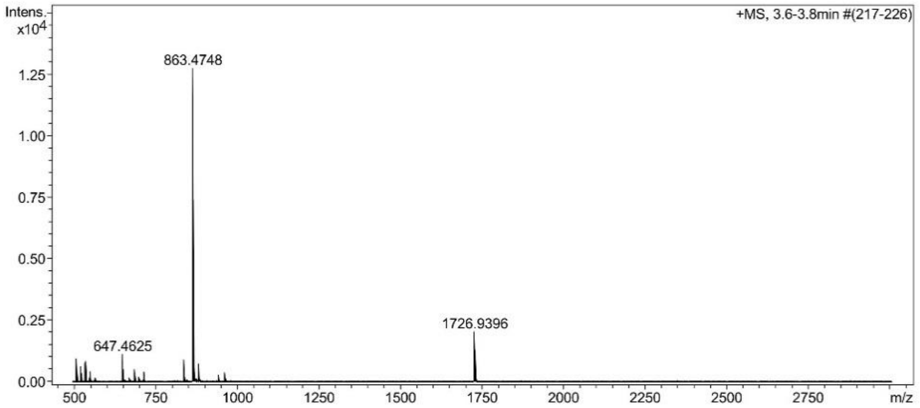

Supplementary Figure 4. Electrospray ionization mass spectrum of **1**.

Synthesis of 2. To a solution of **1** (0.86 g, 1.0 mmol) in acetonitrile (50 mL), K_2CO_3 (0.20 g, 1.5 mmol) was added and the mixture was stirred for 0.5h under nitrogen atmosphere. Then propargyl bromide (0.090 mL, 1.2 mmol) was added. The mixture was heated to 80 °C for 24 h. The cooled reaction mixture was filtered and washed with dichloromethane. After removal of the solvent, the flaxen solid was obtained (**2**, 0.93 g, 0.92 mmol, Yield: 92%). ^{1}H NMR (400 MHz, chloroform-*d*) δ (ppm): 6.83, 6.80, 6.78, 6.77, 6.76, 6.74, 6.72 (m, 10H), 4.45 (s, 2H), 3.89, 3.88, 3.87, 3.86, 3.81, 3.79 (m, 28H), 2.06 (s, 1H), 1.35, 1.33, 1.32, 1.30, 1.30, 1.27, 1.26, 1.24 (m, 27H). ^{13}C NMR (100 MHz, chloroform-*d*) δ (ppm): 150.57, 149.89, 148.79, 129.04, 128.79, 128.69, 128.51, 128.46, 128.38, 128.04, 115.84, 115.32, 115.21, 115.04, 114.93, 78.98, 74.71, 63.93, 63.83, 63.78, 56.42, 30.41, 30.00, 29.83, 29.33, 15.34, 15.25, 15.19, 15.08. HRMS (ESI): $C_{56}H_{69}O_{10}$ $[M]^+$, calcd m/z 901.4885; found m/z 901.4885.

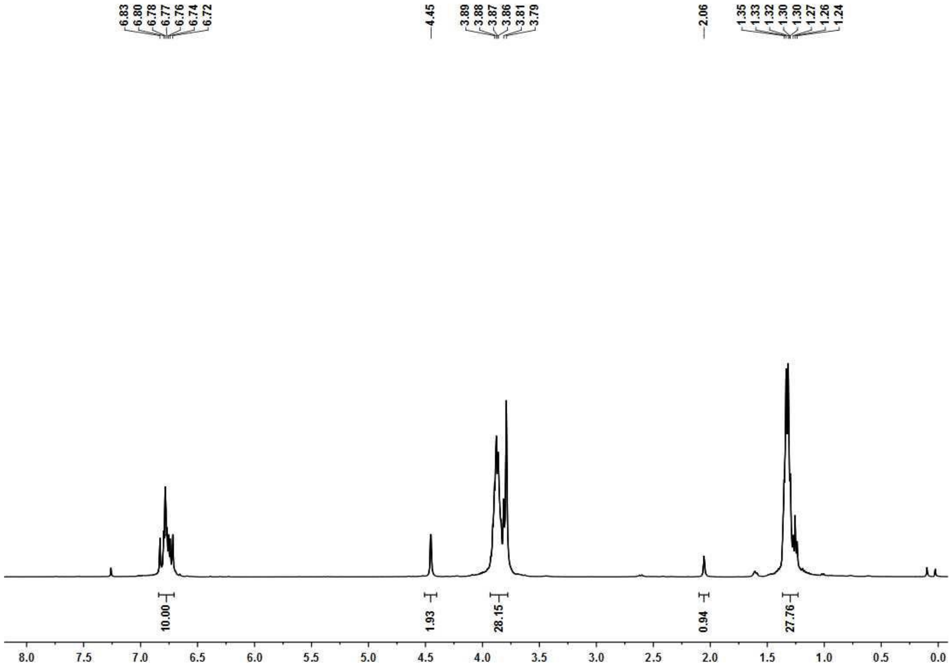

Supplementary Figure 5. ^{1}H NMR spectra (400 MHz, chloroform-*d*, 298 K) of **2**.

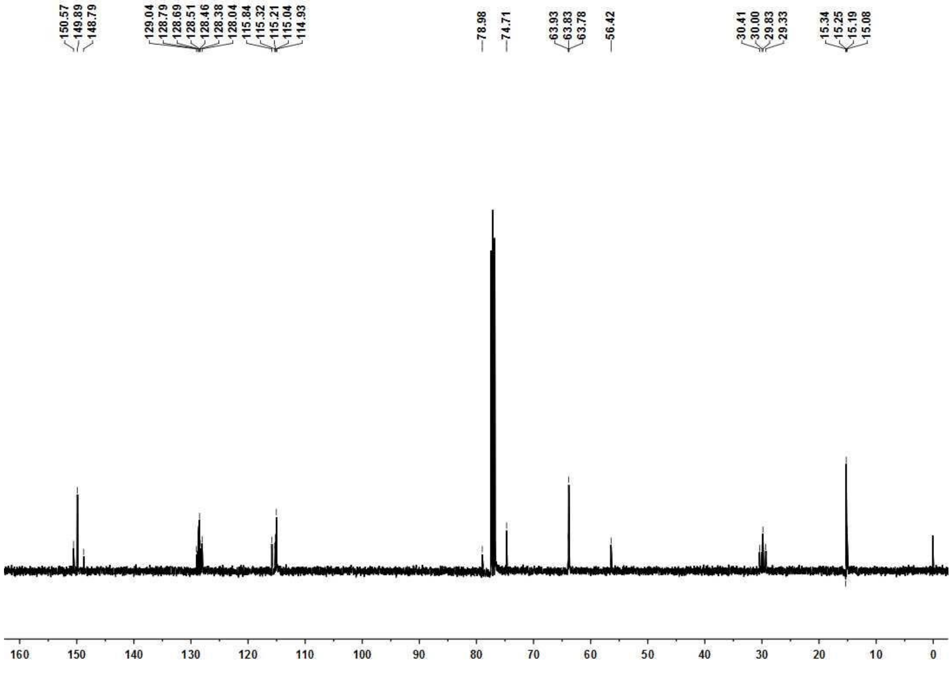

Supplementary Figure 6. ^{13}C NMR spectra (100 MHz, chloroform-*d*, 298 K) of **2**.

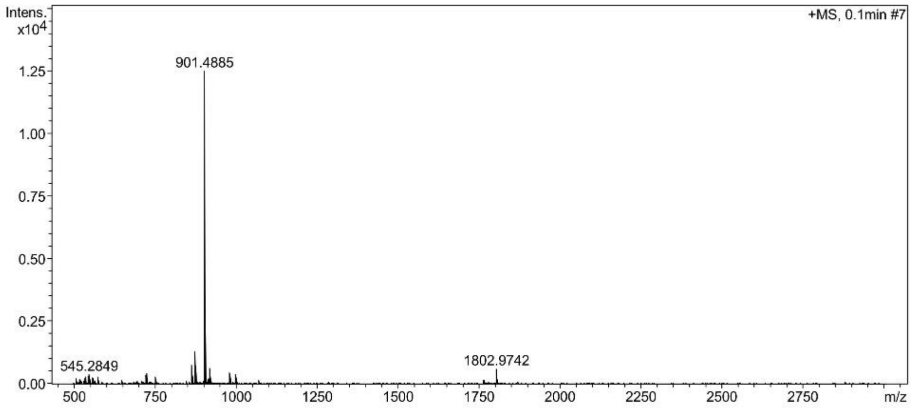

Supplementary Figure 7. Electrospray ionization mass spectrum of **2**.

Synthesis of peptide and purification. All peptides were synthesized by solid-phase synthesis using Fmoc chemistry. Rink amide resin (0.44 mmol g^{-1}) was used as a solid phase to obtain C-terminally amidated peptides. A 20% (v/v) solution of piperidine in *N,N*-dimethylformamide was added to the resin for deprotection. Amino acids were individually coupled to Rink amide resin, followed by the N-terminal conjugation of acetic anhydride. Final cleavage was performed with 10 mL of TFA (85%) / thioanisole (5%)/anisole (5%) / water (5%) for 3 h at room temperature. The peptides were purified by reverse-phase high-performance liquid chromatography (HPLC) using a C8 column (Waters, USA) and a gradient of acetonitrile and deionized (DI) water containing 0.1% (v/v) TFA.

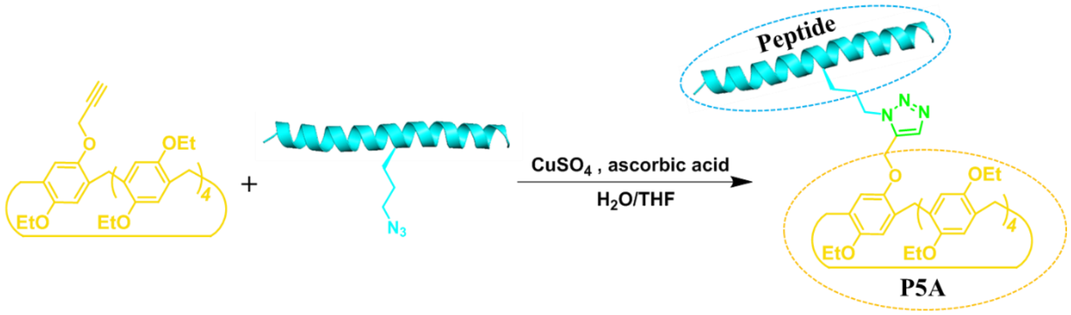

Supplementary Figure 8. Strategy for the preparation of peptide-pillar[5]arene conjugates. Click chemistry provides an orthogonal and productive way to link two large molecules.

Synthesis of peptide-pillar[5]arene conjugates. Under a nitrogen atmosphere, **2** in THF was added to a solution of peptide, copper sulphate pentahydrate and sodium ascorbic in water. The reaction mixture was stirred for 4h at room temperature. Then the mixture was filtered and washed with water. After removal of THF, the conjugates were purified by reverse-phase high-performance liquid chromatography (HPLC) using a C8 column (Waters, USA) and a gradient

of acetonitrile and deionized (DI) water containing 0.1% (v/v) TFA. Molecular weights (MWs) of peptides were analyzed by high-resolution mass spectrometry.

Supplementary Table 1. Sequence of peptide and molecular weight of peptide-pillar[5]arene conjugates.

Compound	Peptide sequence	Molecular weight	
		Calculated	Measured
P1P5A	RGDSK(N_3)EEEE	2045.58	2045.972
P2P5A	GGDSK(N_3)EEEE	1946.44	1946.870
P3P5A	RGESK(N_3)EEEE	2059.40	2059.967
P4P5A	RGDSK(N_3)EEEEK(NBD)	2336.84	2337.067

a

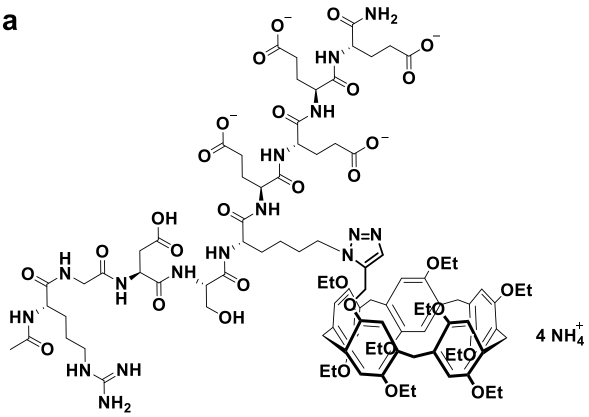

P1P5A

b

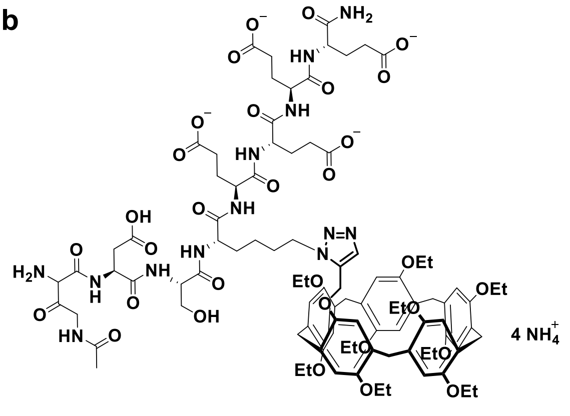

P2P5A

c

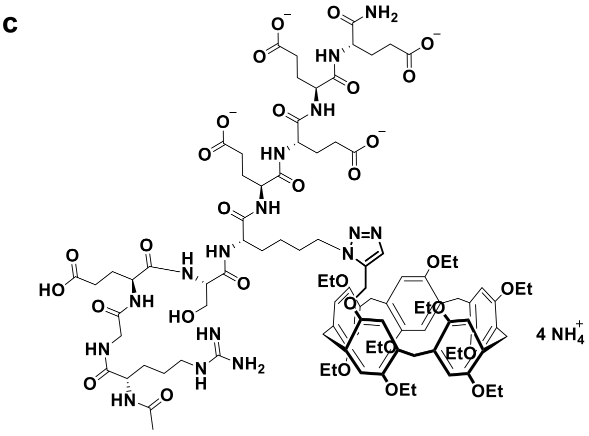

P3P5A

d

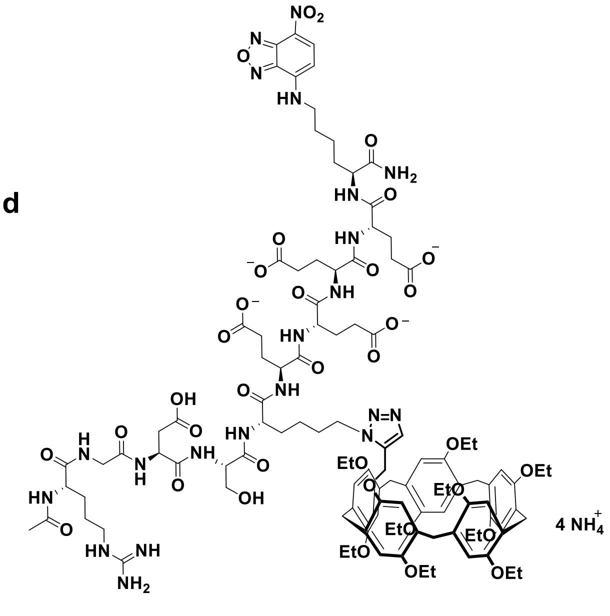

P4P5A

Supplementary Figure 9. Chemical structure of peptide-pillar[5]arene conjugates.

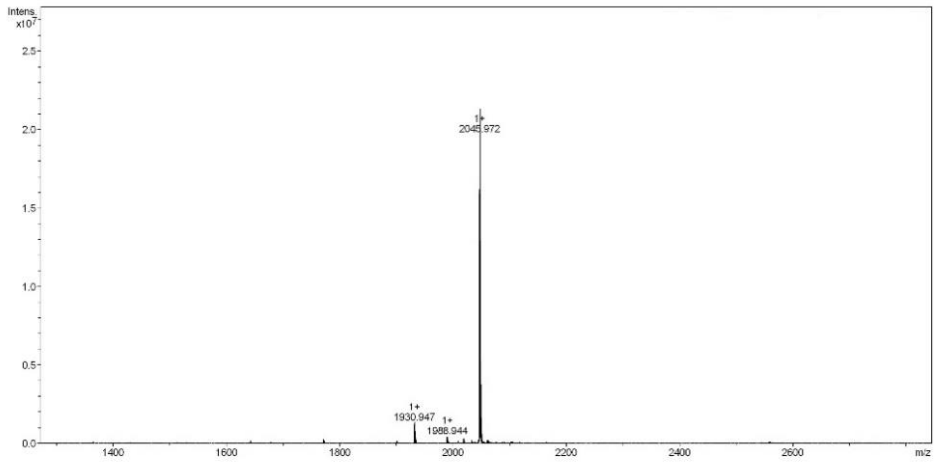

Supplementary Figure 10. High-resolution mass spectrum of P1P5A.

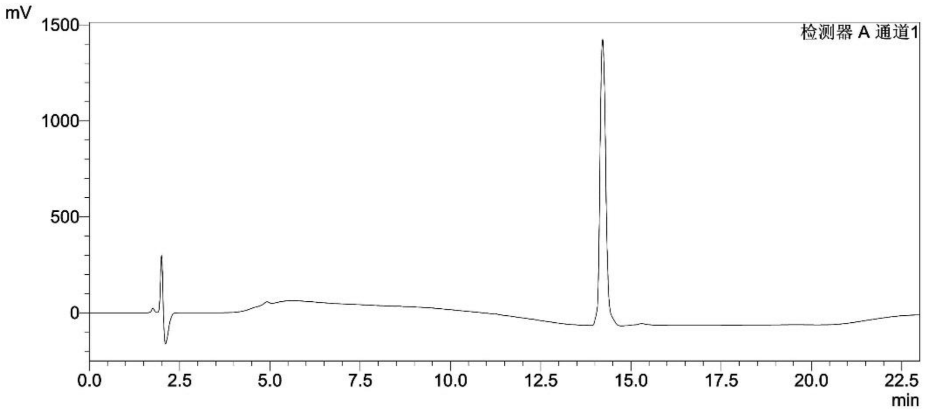

Supplementary Figure 11. RP-HPLC of P1P5A.

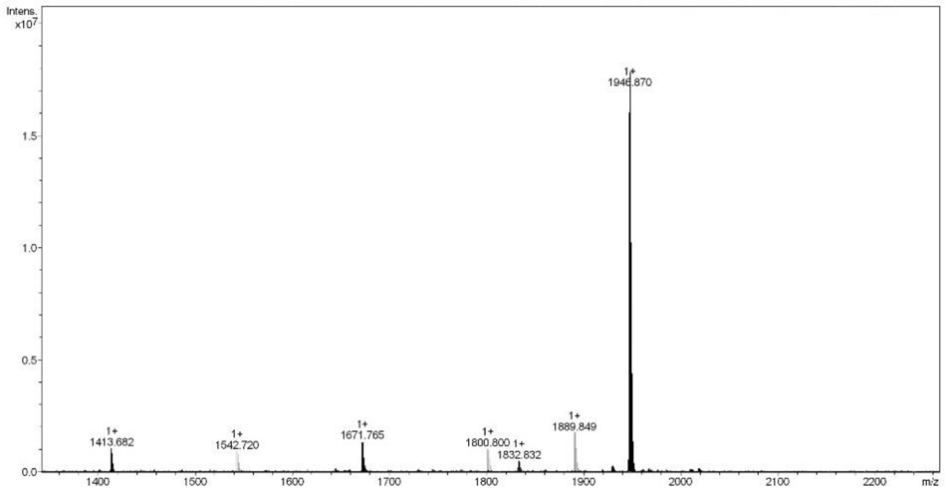

Supplementary Figure 12. High-resolution mass spectrum of P2P5A.

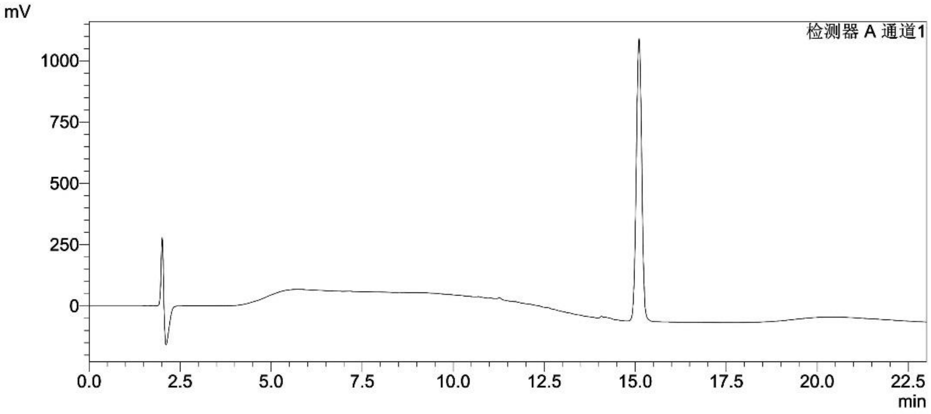

Supplementary Figure 13. RP-HPLC of P2P5A.

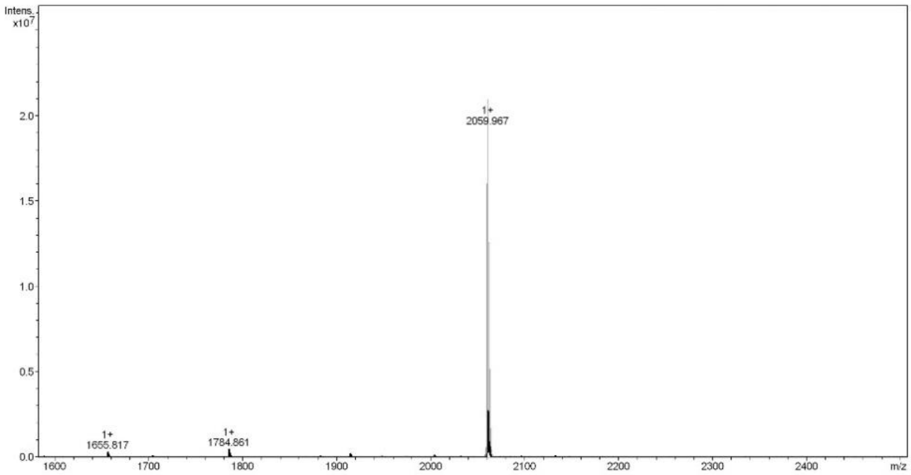

Supplementary Figure 14. High-resolution mass spectrum of P3P5A.

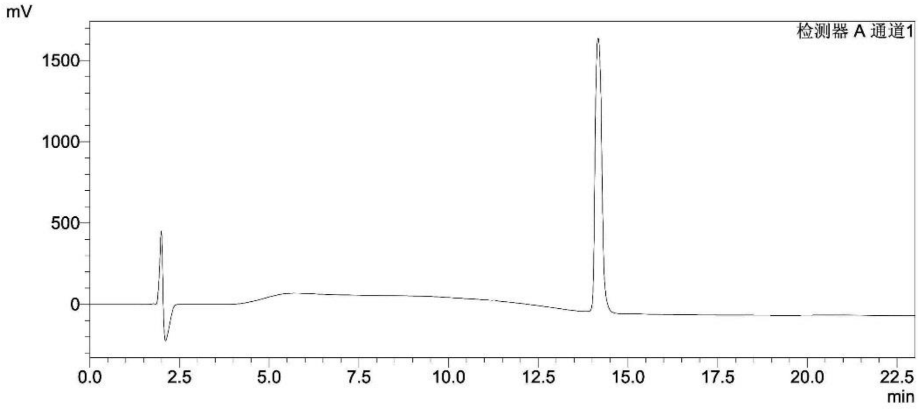

Supplementary Figure 15. RP-HPLC of P3P5A.

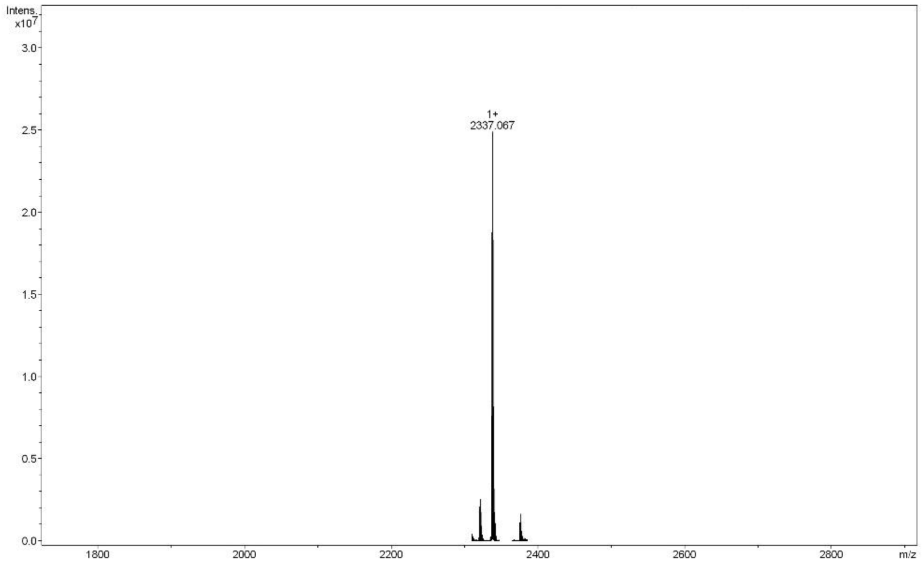

Supplementary Figure 16. High-resolution mass spectrum of P4P5A.

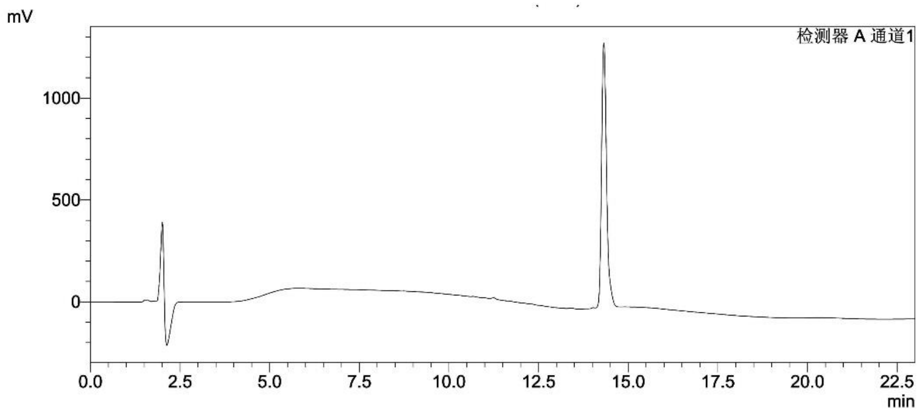

Supplementary Figure 17. RP-HPLC of P4P5A.

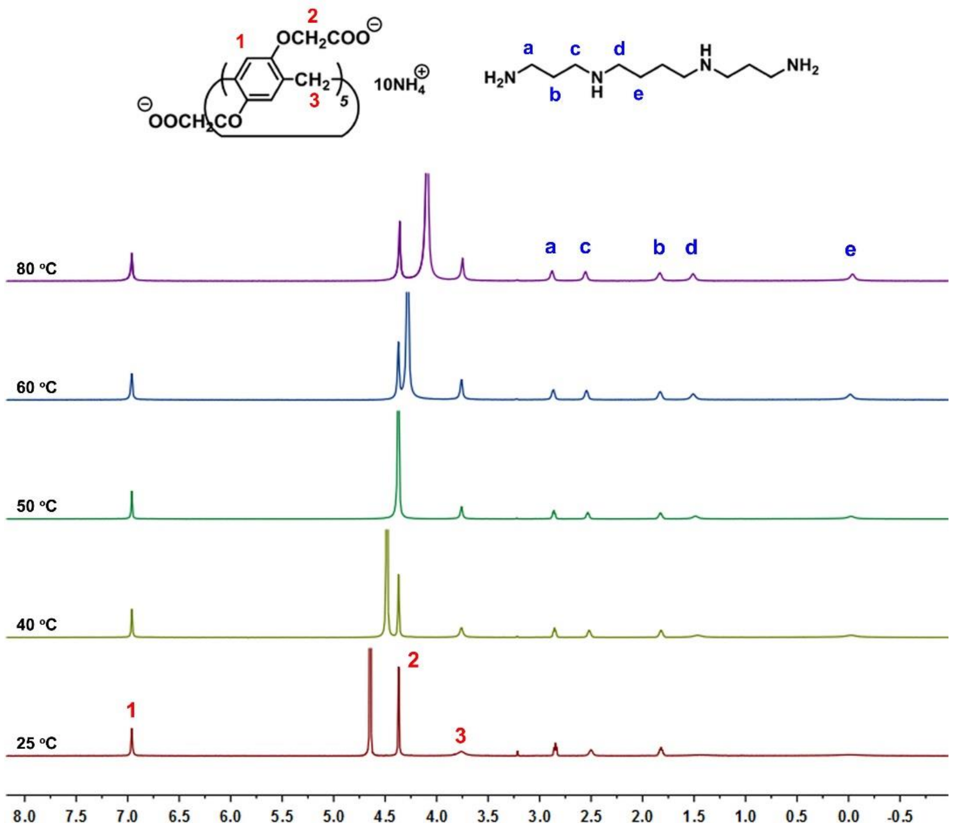

Supplementary Figure 18. Variable-temperature ^{1}H NMR spectra (500 MHz, D_2O) of spm (3.0 mM) and CP5A (3.0 mM). The temperatures were 25, 40, 50, 60 and 80 °C.

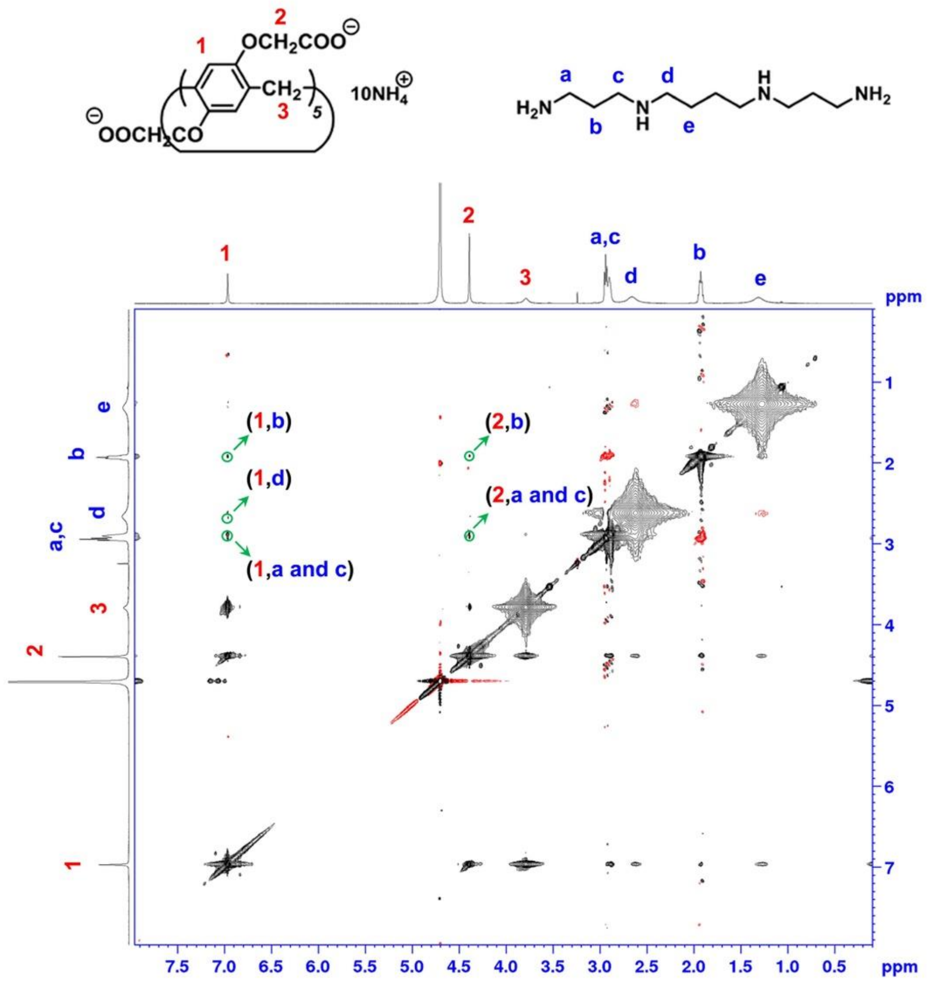

Supplementary Figure 19. 2D NOESY (500 MHz, 298 K, D_2O) of spm (15.0 mM) and CP5A (3.0 mM) with a mixing time of 600ms.

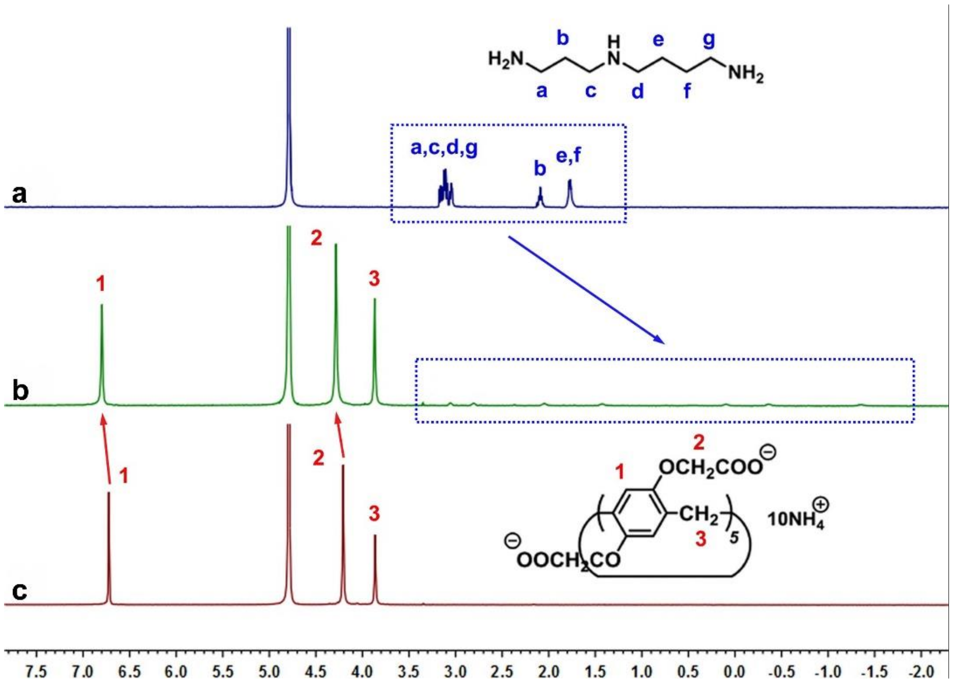

Supplementary Figure 20. ^{1}H NMR spectra (500 MHz, 298 K, D_2O) of **a** spd (3.0 mM), **b** spd (3.0 mM) + CP5A (3.0 mM) and **c** CP5A (3.0 mM).

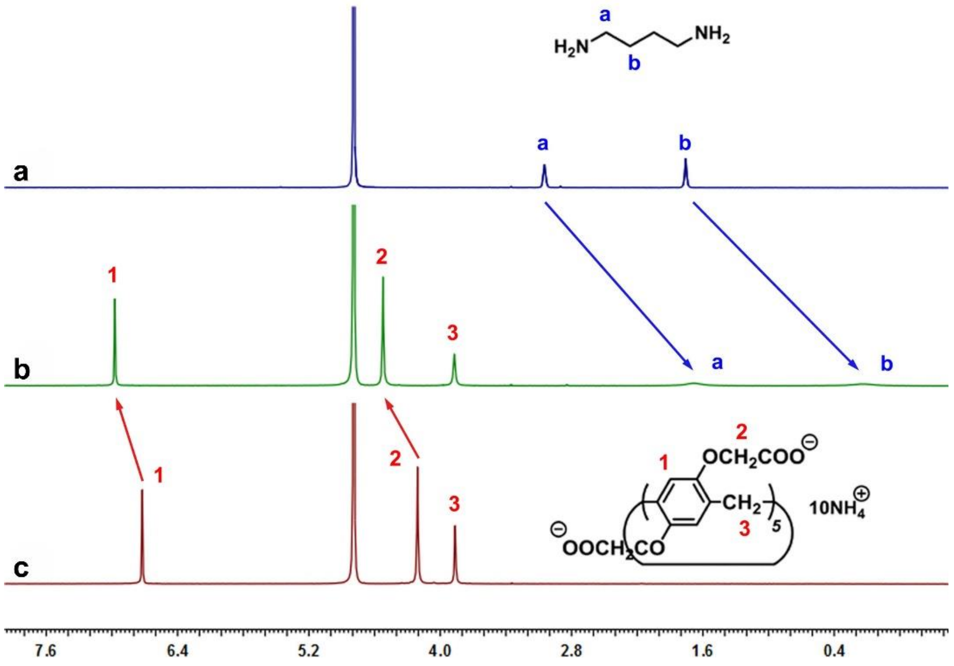

Supplementary Figure 21. ^{1}H NMR spectra (500 MHz, 298 K, D_2O) of **a** put (3.0 mM), **b** put (3.0 mM) + CP5A (3.0 mM) and **c** CP5A (3.0 mM).

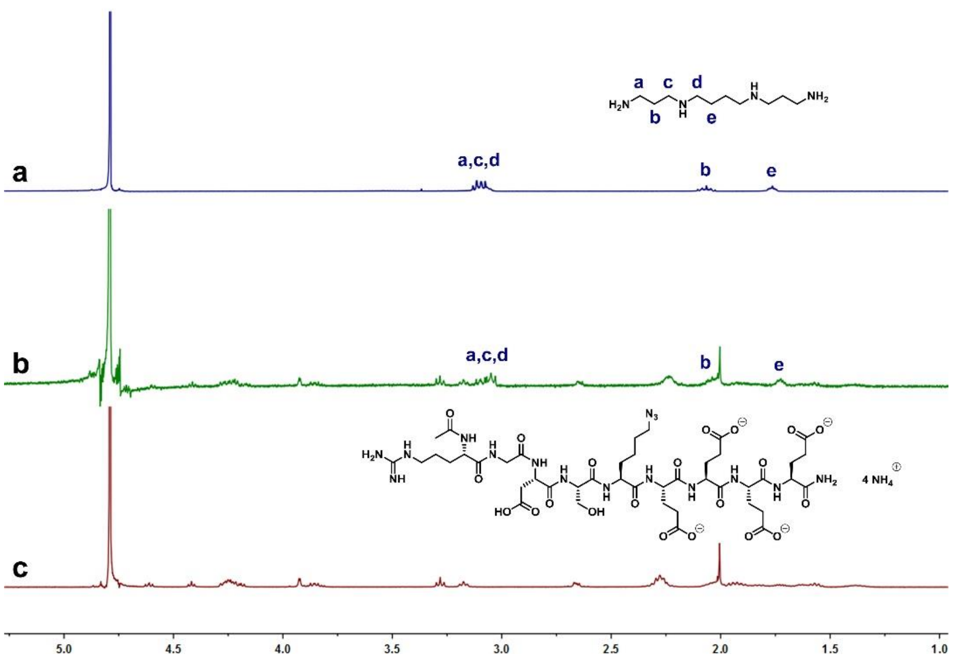

Supplementary Figure 22. ^{1}H NMR spectra (400 MHz, 298 K, D_2O) of **a** spm (3.0 mM), **b** spm (3.0 mM) + P1 (3.0 mM) and **c** P1 (3.0 mM). No precipitate was observed by mixing P1 moiety and spm. In the presence of P1, the spm signals exhibit small shifts (Δδ = −0.02 ~ −0.05 ppm for protons a~e), suggesting their weak interactions.

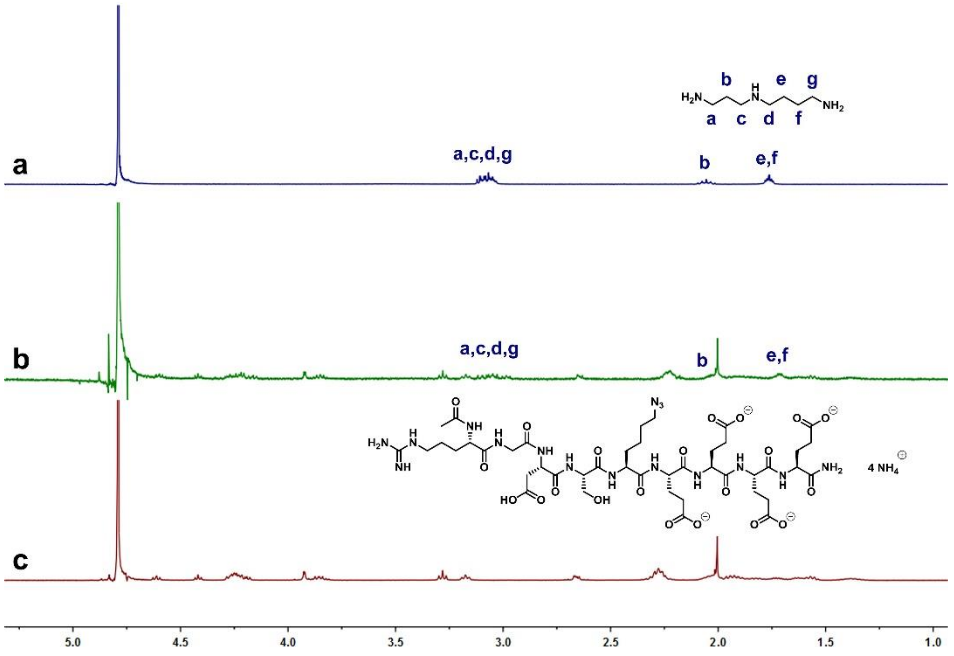

Supplementary Figure 23. ^{1}H NMR spectra (400 MHz, 298 K, D_2O) of **a** spd (3.0 mM), **b** spd (3.0 mM) + P1 (3.0 mM) and **c** P1 (3.0 mM). No precipitate was observed by mixing P1 moiety and spd. In the presence of P1, the spd signals exhibit small shifts (Δδ = −0.02 ~ −0.05 ppm for protons a~g), suggesting their weak interactions.

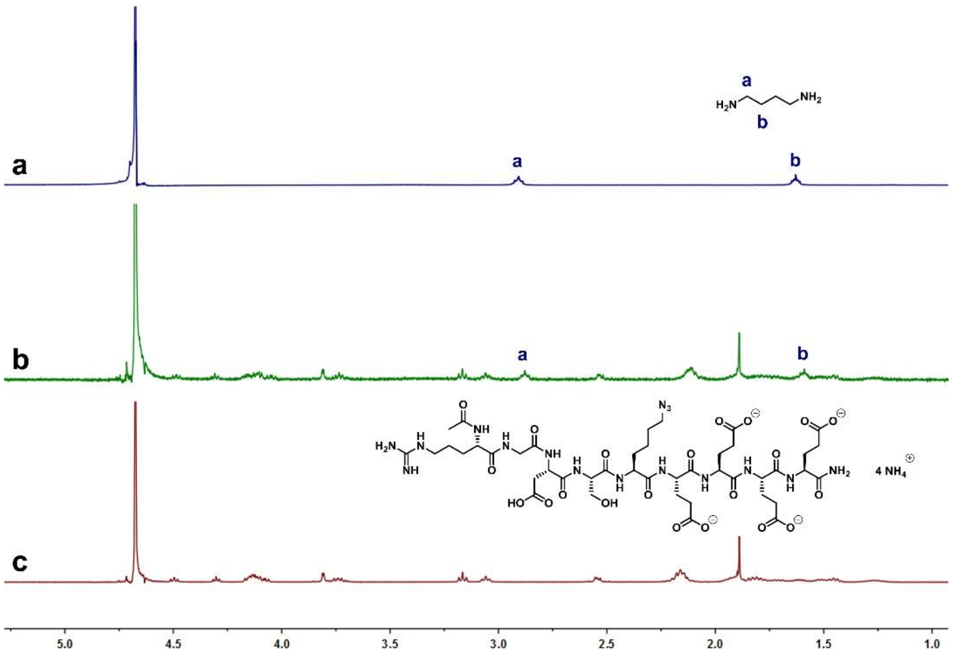

Supplementary Figure 24. ^{1}H NMR spectra (400 MHz, 298 K, D_2O) of **a** put (3.0 mM), **b** put (3.0 mM) + P1 (3.0 mM) and (c) P1 (3.0 mM). No precipitate was observed by mixing P1 moiety and put. In the presence of P1, the put signals exhibit small shifts (Δδ = −0.03 ~ −0.04 ppm for protons a~b), suggesting their weak interactions.

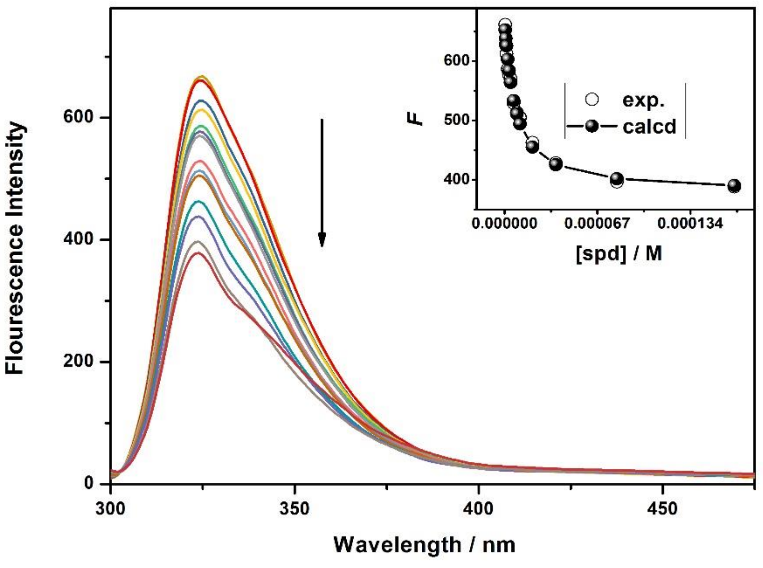

Supplementary Figure 25. Fluorescence spectra of P1P5A (2.46 × 10^{-6} M) in aqueous phosphate buffer solution (pH 7.4) with different concentrations of spd at 298 K, inset: the nonlinear least-squares analysis to calculate the K_a value.

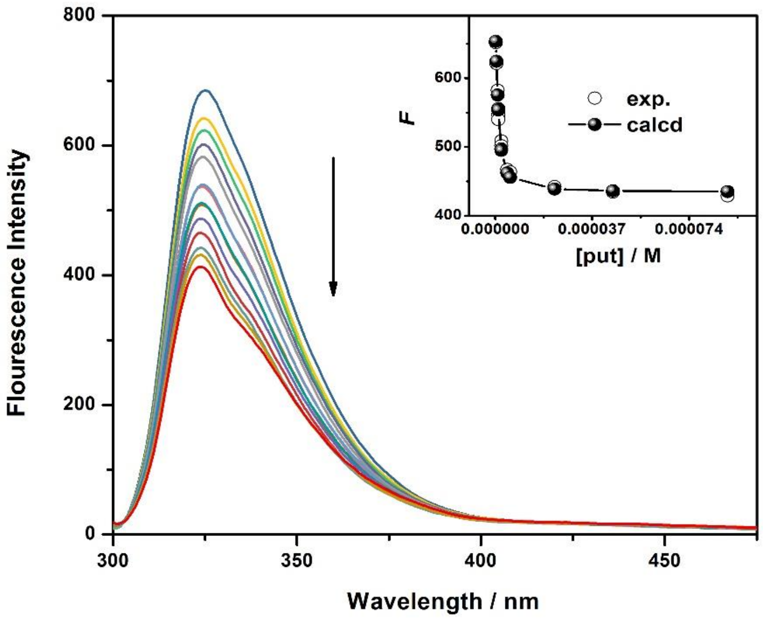

Supplementary Figure 26. Fluorescence spectra of P1P5A (2.46 × 10^{-6} M) in aqueous phosphate buffer solution (pH 7.4) with different concentrations of put at 298 K, inset: the nonlinear least-squares analysis to calculate the K_a value.

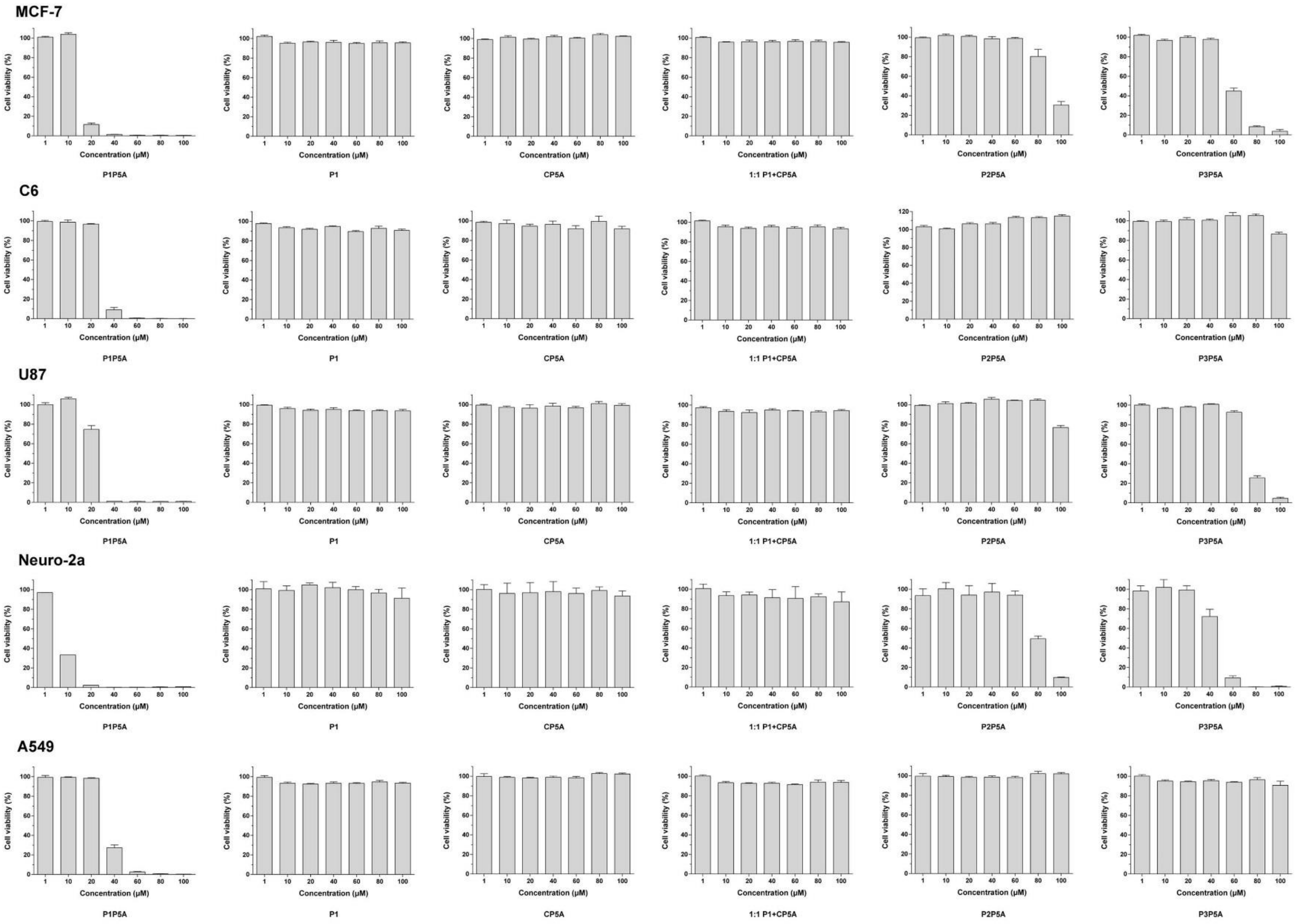
MCF-7
Cell viability (%)
Concentration (μM)
P1P5A
P1
CP5A
1:1 P1+CP5A
P2P5A
P3P5A
C6
P1P5A
P1
CP5A
1:1 P1+CP5A
P2P5A
P3P5A
U87
P1P5A
P1
CP5A
1:1 P1+CP5A
P2P5A
P3P5A
Neuro-2a
P1P5A
P1
CP5A
1:1 P1+CP5A
P2P5A
P3P5A
A549
P1P5A
P1
CP5A
1:1 P1+CP5A
P2P5A
P3P5A

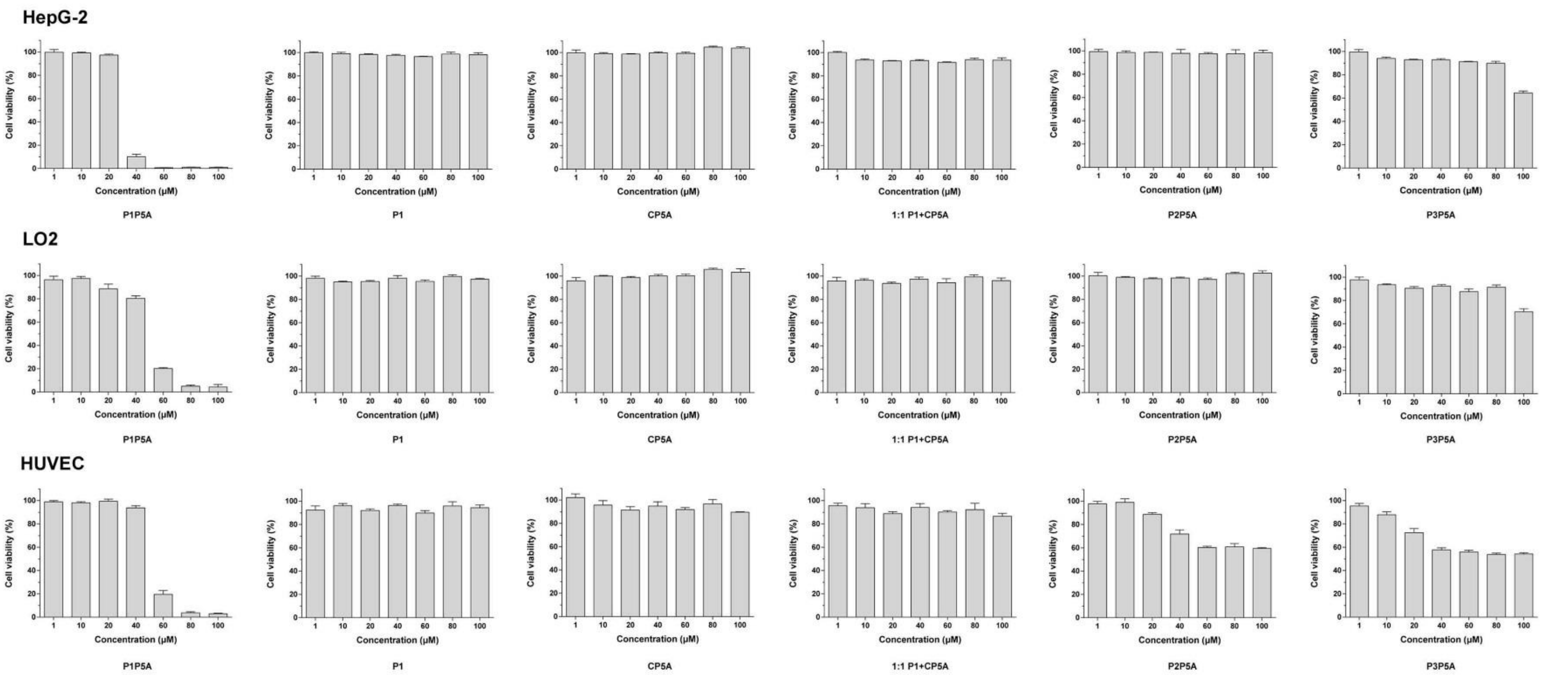

Supplementary Figure 27. Cytotoxicity of compounds. MCF-7 (human breast adenocarcinoma cells), C6 (rat glioma cells), U87 (human glioma cells), Neuro-2a (mouse neuroblastoma N2a cells), A549 ((lung adenocarcinoma epithelia cells), HepG-2 (liver hepatocellular carcinoma), LO2 (human normal liver cells) and HUVEC (human umbilical vein endothelial cells) were treated with various concentrations (1-100 μM) of each compound for 48h. Cell death was then measured by using a CCK-8 assays (n=5, mean ± SD).

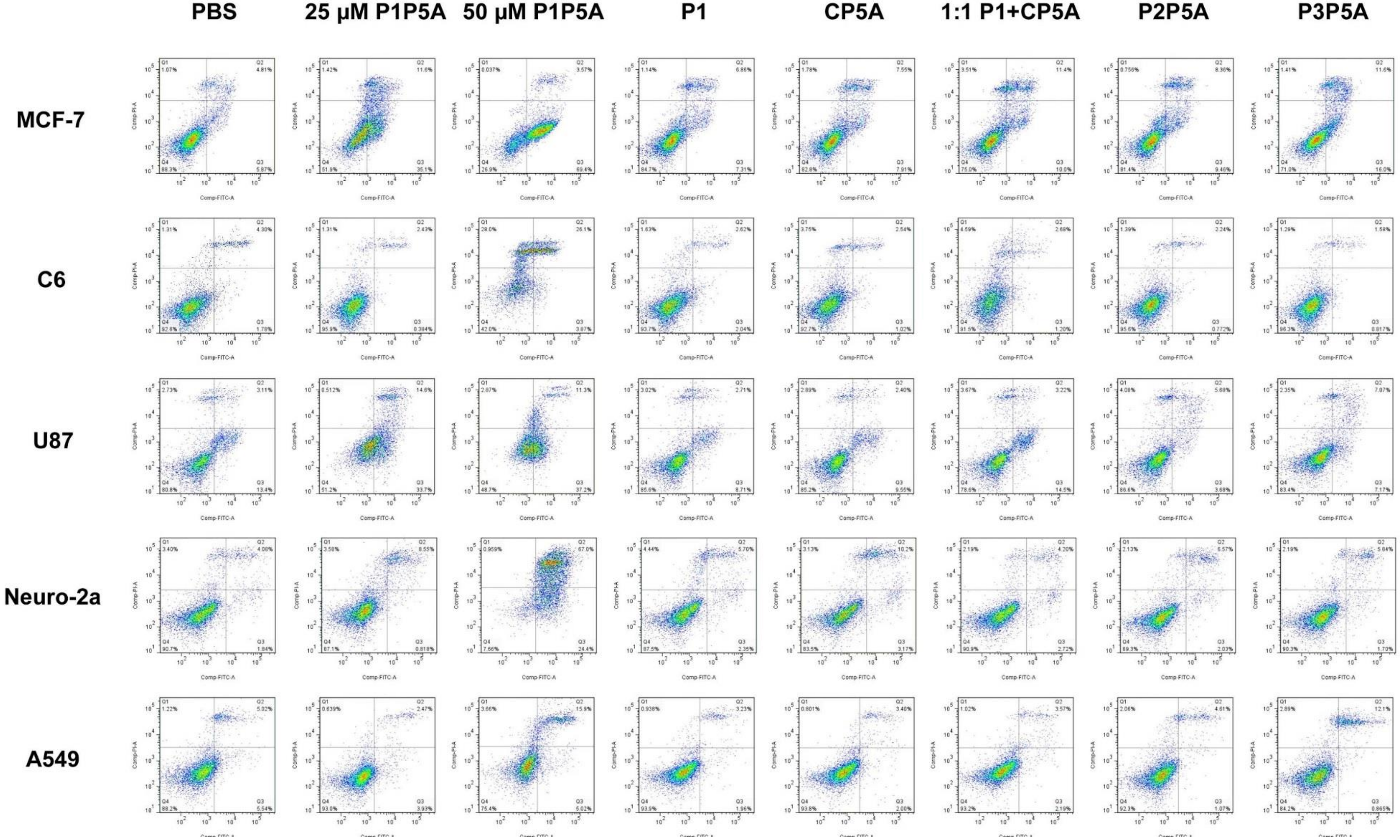
PBS
25 μM P1P5A
50 μM P1P5A
P1
CP5A
1:1 P1+CP5A
P2P5A
P3P5A
MCF-7
C6
U87
Neuro-2a
A549
Comp-PI-A
Comp-FITC-A

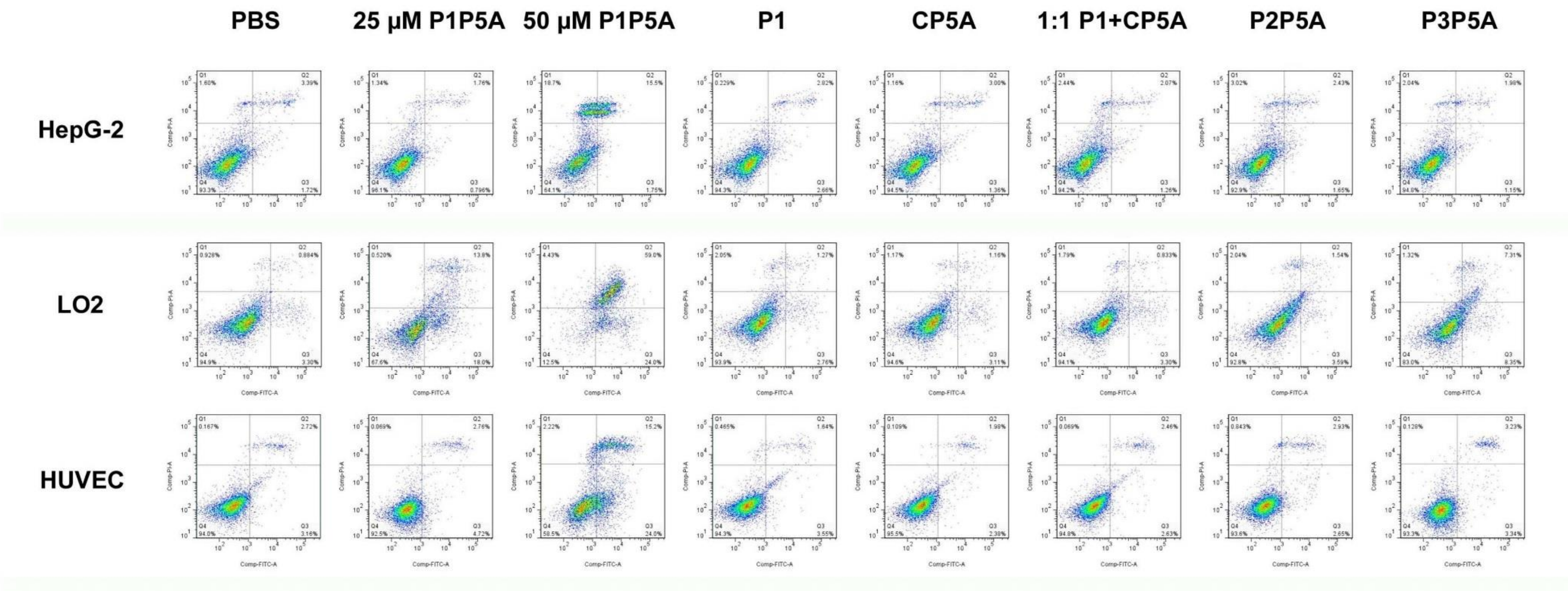

Supplementary Figure 28. Synthetic conjugates induce apoptosis. Flow cytometry of all cells first treated with above compounds under study for 48h and then stained with fluorescein-Annexin V and PI (allowing analysis of Annexin V binding *versus* PI uptake).

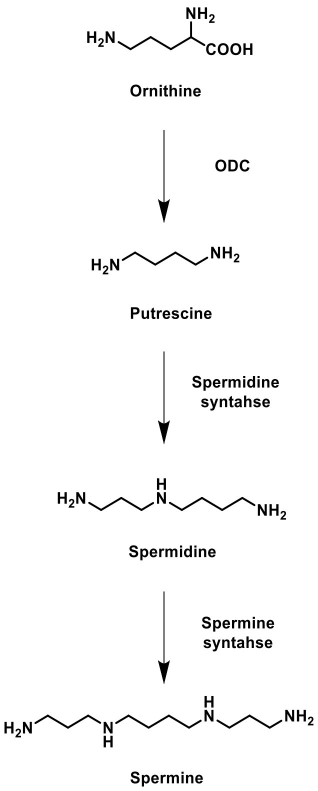

Supplementary Figure 29. Schematic of polyamines biosynthesis pathway.

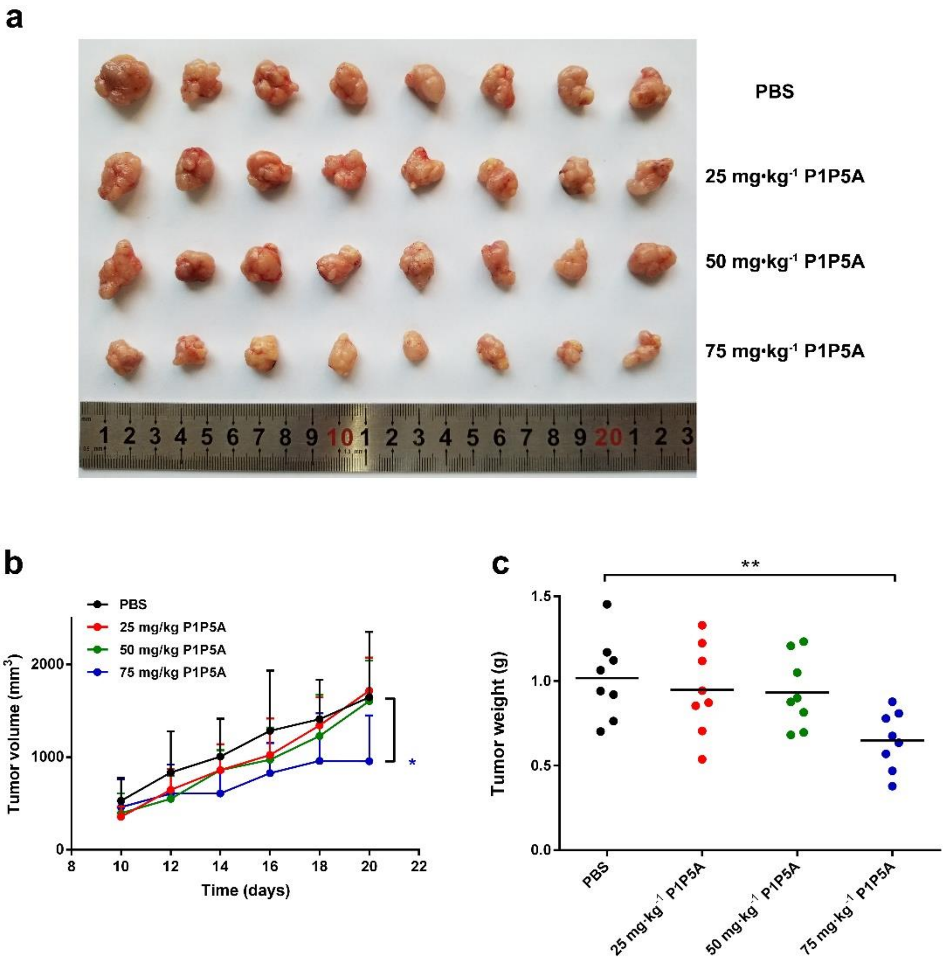

Supplementary Figure 30. *In vivo* antitumor experiments. **a** Images of tumors excised from MCF-7 xenograft nude mice treated with PBS and three doses of P1P5A; **b** tumor growth curves of the MCF-7 tumor model in the groups treated with PBS and three doses of P1P5A, mean ± SD (n = 8 mice per groups); **c** normalized tumor weights at 10 days after treatment with PBS and three doses of P1P5A, mean ± SD (n = 8 mice per group). Significant differences were assessed in **b** and **c** using the t-test. *p < 0.05, **p < 0.01. The normalized tumor weight of PBS group (1.02 ± 0.24 g) was 57 % higher than that of P1P5A group of 75 mg/kg (0.65 ± 0.17 g).

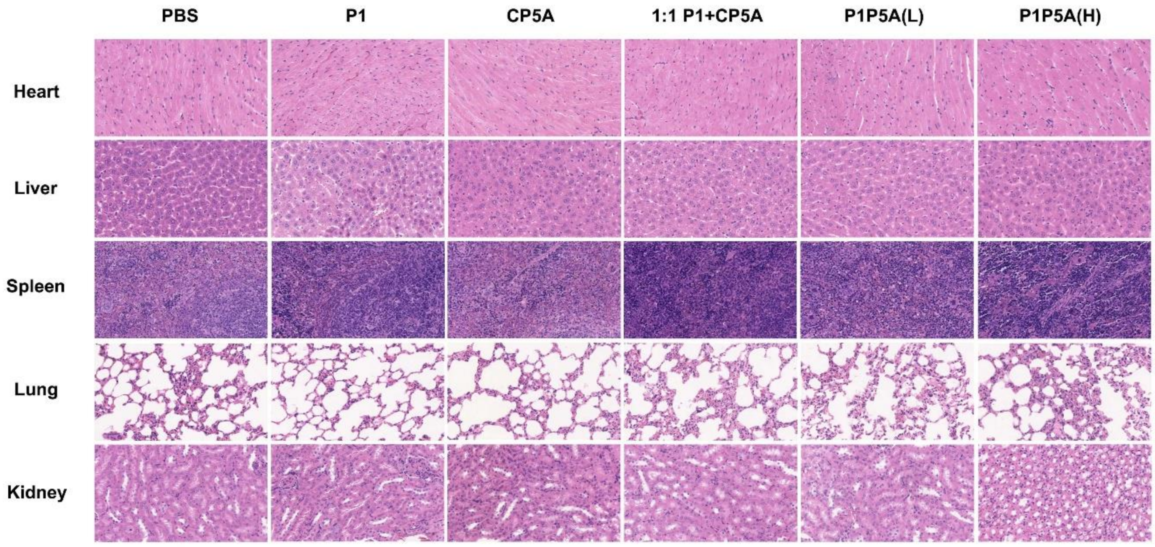

Supplementary Figure 31. Histopathologic analysis of major organs (heart, liver, spleen, lung and kidneys) stained with H&E at 10 days after treatment with PBS, P1, CP5A, 1:1 P1+CP5A, P1P5A (L), and P1P5A (H).

References

1 Li, C. *et al.* Complexation of 1,4-bis(pyridinium)butanes by negatively charged carboxylatopillar[5]arene. *J. Org. Chem.* **76**, 8458-8465 (2011).
